# Supplementary material for: Role of specialized composition of SWI/SNF complexes in prostate cancer lineage plasticity
Source: Nat Commun. 2020 Nov 3;11:5549. doi: 10.1038/s41467-020-19328-1 (PMC7642293; doi:10.1038/s41467-020-19328-1)
Supplement: Supplementary file 1 — Supplementary Information [file 41467_2020_19328_MOESM1_ESM.pdf]

Supplementary Information

Role of Specialized Composition of SWI/SNF Complexes in Prostate Cancer Lineage Plasticity

Joanna Cyrta[1,2 *], Anke Augspach[1 *], Maria Rosaria De Filippo[3,4], Davide Prandi[5], Phillip Thienger[1], Matteo Benelli[5,6], Victoria Cooley[7], Rohan Bareja[2,8], David Wilkes[2], Sung-Suk Chae[9], Paola Cavaliere[10], Noah Dephoure[10,11], Anne-Christine Uldry[12], Sophie Braga Lagache[12], Luca Roma[4], Sandra Cohen[9], Muriel Jaquet[1], Laura P. Brandt[1], Mohammed Alshalalfa[13], Loredana Puca[14], Andrea Sboner[2,8,15,16], Felix Feng[12], Shangqian Wang[17], Himisha Beltran[14,18], Tamara Lotan[19,20,21], Martin Spahn[22,23], Marianna Kruithof-de Julio[1,3,24], Yu Chen[14], Karla V. Ballman[7], Francesca Demichelis[2,5], Salvatore Piscuoglio[4,25,26 #] and Mark A. Rubin[1,27,28 #§].

* these authors contributed equally (first co-authorship)
# these authors jointly supervised this work
§ correspondences to mark.rubin@dbmr.unibe.ch

1. Department for BioMedical Research, University of Bern, 3010 Bern, Switzerland
2. The Caryl and Israel Englander Institute for Precision Medicine, Weill Cornell Medicine, New York, NY 10021, USA
3. Department for BioMedical Research, Urology Research Laboratory, University of Bern, 3010 Bern, Switzerland
4. Institute of Pathology and Medical Genetics, University Hospital Basel, University of Basel, Basel, Switzerland
5. Department of Cellular, Computational and Integrative Biology (CIBIO), University of Trento, Trento, Italy
6. Bioinformatics Unit, Hospital of Prato, Prato, Italy
7. Department of Healthcare Policy & Research, Division of Biostatistics and Epidemiology, Weill Cornell Medicine, New York, NY 10021, USA
8. Institute for Computational Biomedicine, Weill Cornell Medicine, New York, NY 10021, USA
9. Department of Laboratory Medicine and Pathology, Weill Cornell Medicine, New York, NY 10021, USA
10. Meyer Cancer Center, Weill Cornell Medicine, New York, NY 10021, USA
11. Department of Biochemistry, Weill Cornell Medicine, New York, NY 10021, USA
12. Proteomics Mass Spectrometry Core Facility, University of Bern, 3010 Bern, Switzerland
13. Department of Radiation Oncology, Helen Diller Family Comprehensive Cancer Center, University of California at San Francisco, San Francisco, CA, USA
14. Department of Medicine, Division of Medical Oncology, Weill Cornell Medicine, New York, NY, USA
15. HRH Prince Alwaleed Bin Talal Bin Abdulaziz Alsaud Institute for Computational Biomedicine, Weill Cornell Medicine, New York, NY 10021, USA
16. Meyer Cancer Center, Weill Cornell Medicine, New York, NY 10065, USA
17. Human Oncology and Pathogenesis Program and Department of Medicine, Memorial Sloan-Kettering Cancer Center, New York, NY 10065, USA
18. Department of Medical Oncology, Dana Farber Cancer Institute, Boston, MA, USA
19. Department of Urology, Johns Hopkins University School of Medicine, Baltimore, Maryland, USA.
20. Department of Pathology, Johns Hopkins University School of Medicine, Baltimore, Maryland, USA
21. Department of Oncology, Johns Hopkins University School of Medicine, Baltimore, Maryland, USA
22. Lindenhofspital Bern, Prostate Center Bern, 3012 Bern, Switzerland
23. Department of Urology, Essen University Hospital, University of Duisburg-Essen, Essen, Germany
24. Department of Urology, Inselspital, 3010 Bern, Switzerland
25. Visceral Surgery Research Laboratory, Clarunis, Department of Biomedicine, University of Basel, Basel, Switzerland
26. Clarunis Universitäres Bauchzentrum Basel, 4002 Basel, Switzerland
27. Inselspital, 3010 Bern, Switzerland
28. Bern Center for Precision Medicine, 3010 Bern, Switzerland

Supplementary Figure 1 (related to Fig 1)

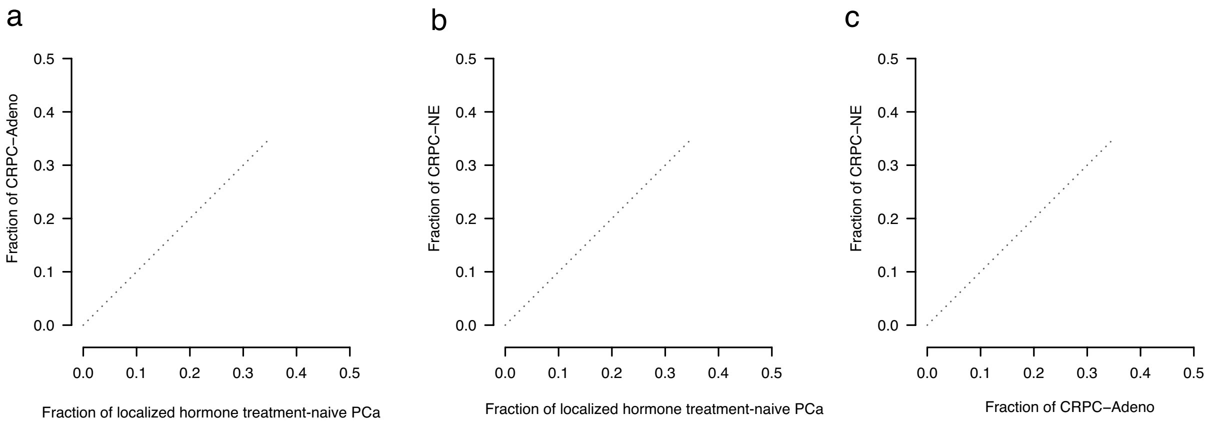

Graphs showing the fraction of cases with loss-of-heterozygosity (LOH) for each SWI/SNF gene across PCa disease states in patient samples. Each point on the graph represents a SWI/SNF gene. The graphs compare the fraction of cases with LOH for each gene in CRPC-Adeno *versus* localized hormone treatment-naïve PCa **(a)**, in CRPC-NE *versus* localized hormone treatment-naïve PCa **(b)** and in CRPC-NE *versus* CRPC-Adeno **(c).**

Supplementary Figure 2 (related to Fig 1)

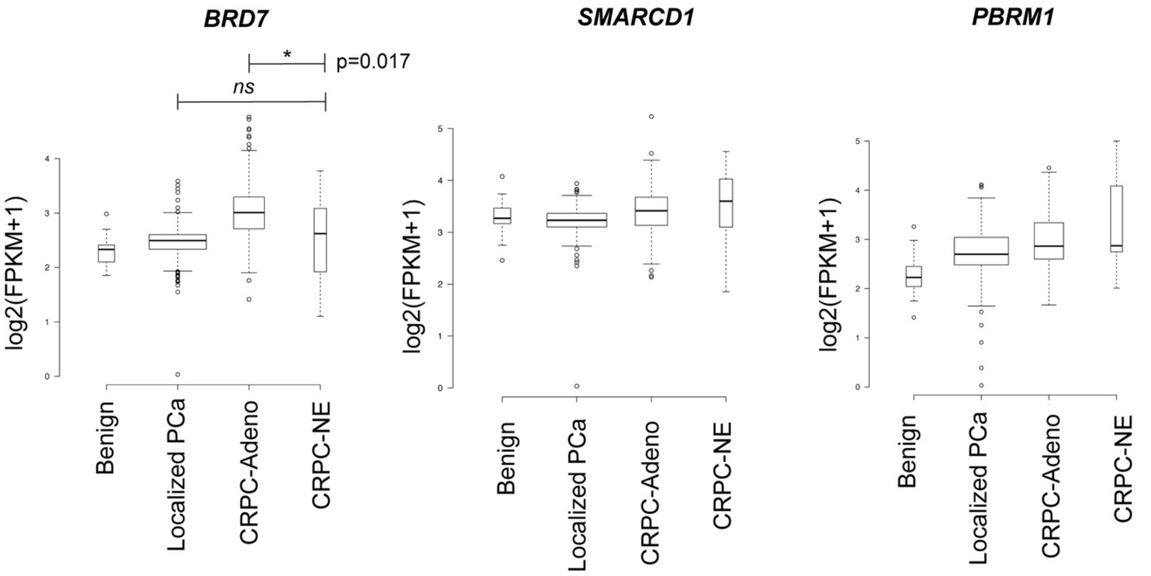

BRD7, SMARCD1 and PBRM1 **gene expression ratios (RNA-seq) across PCa disease states in patient samples.** Expression was compared using the Mann-Whitney Wilcoxon test. The box plots represent the median values and the lower and upper interquartile range (IQR); the upper whisker = min(max(x), Q3 + 1.5 × IQR) and lower whisker = max(min(x), Q1 − 1.5 × IQR), and the outliers are plotted as individual points. Abbreviations: Benign: benign prostatic tissue (n=32); PCa: localized, hormone treatment-naïve prostatic adenocarcinoma (n=400); CRPC-Adeno: Castration resistant prostate cancer, adenocarcinoma subtype (n=120); CRPC-NE: Castration resistant prostate cancer, neuroendocrine subtype (n=20).

Supplementary Figure 3 (related to Fig 1)

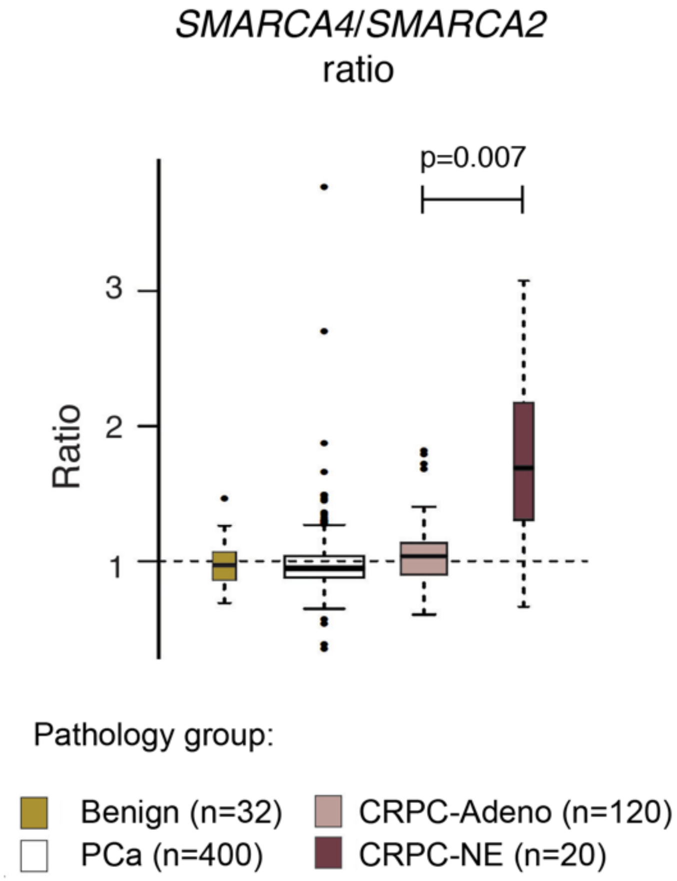

***SMARCA4/SMARCA2* gene expression ratios (RNA-seq) across PCa disease states in patient samples.** Expression of the ratios between groups was compared using the Mann-Whitney Wilcoxon test. The box plots represent the median values and the lower and upper interquartile range (IQR); the upper whisker = min(max(x), Q3 + 1.5 × IQR), the lower whisker = max(min(x), Q1 − 1.5 × IQR), and the outliers are plotted as individual points. Abbreviations: Benign: benign prostatic tissue; PCa: localized, hormone treatment-naïve prostatic adenocarcinoma; CRPC-Adeno: Castration resistant prostate cancer, adenocarcinoma subtype; CRPC-NE: Castration resistant prostate cancer, neuroendocrine subtype.

Supplementary Figure 4 (related to Fig 1)

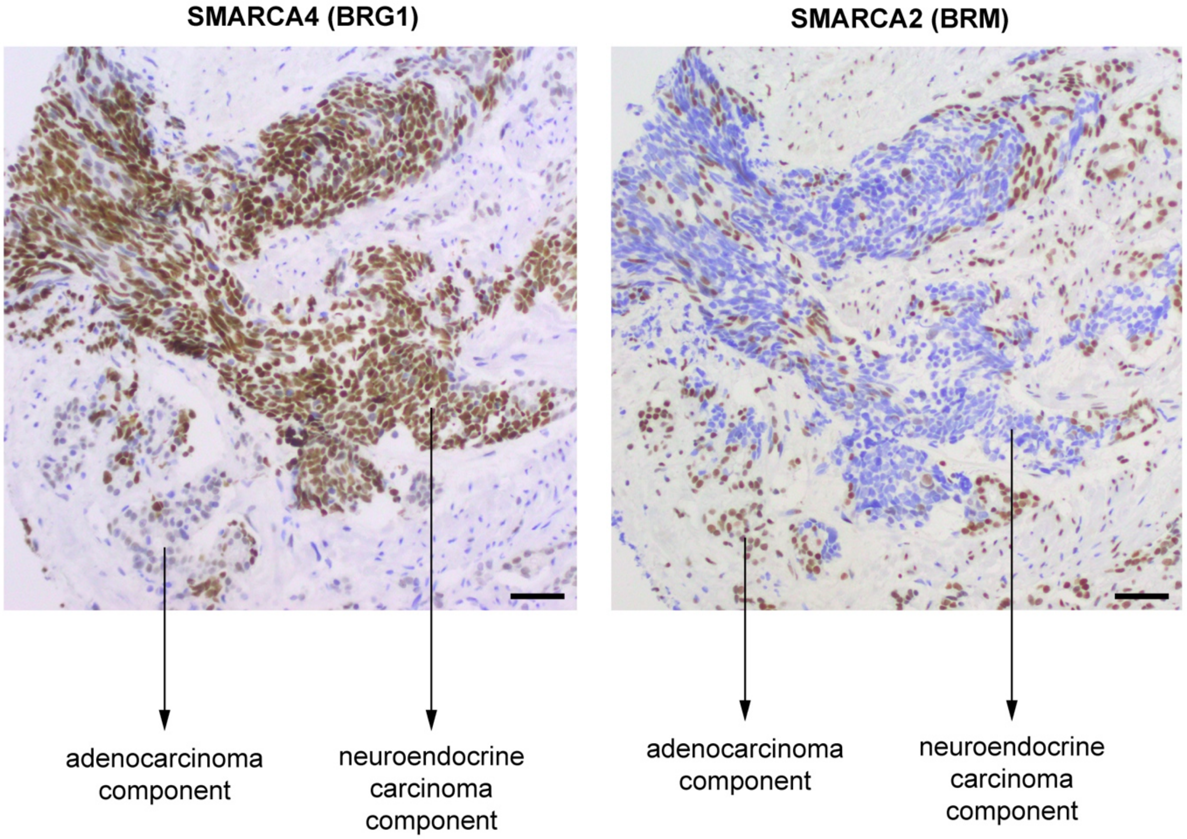

Immunohistochemistry for *SMARCA4* (BRG1) and *SMARCA2* (BRM) in a patient FFPE sample of mixed PCa with a neuroendocrine and an adenocarcinoma component. The case illustrates intra-tumor heterogeneity in expression levels of both catalytic paralogues. Scale bars, 50 μm. Microphotographs show one representative case. In total, three cases of mixed PCa from three different patients were tested by IHC (including the case shown here), with concordant results.

Supplementary Figure 5 (related to Fig 1)

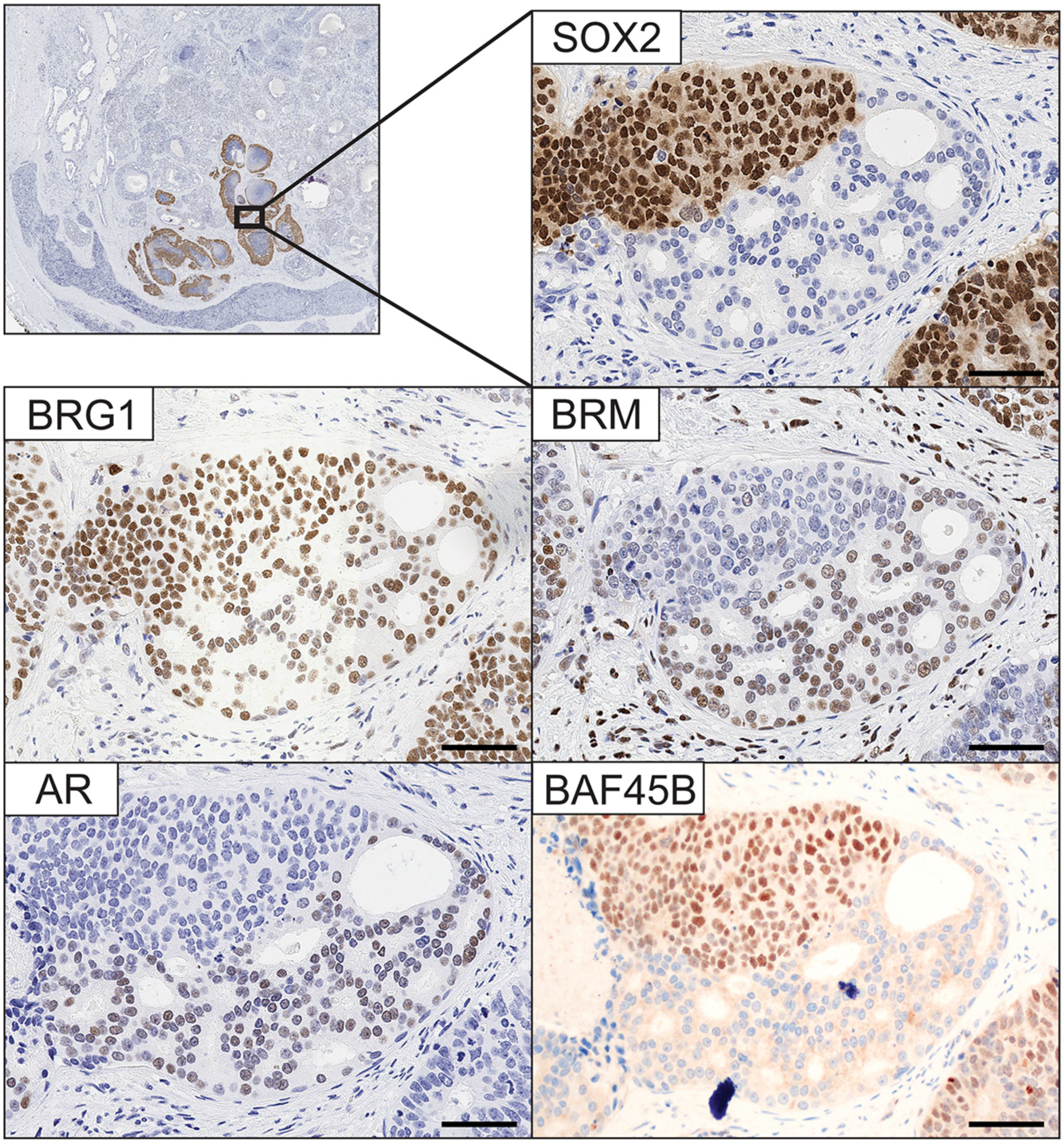

Immunohistochemistry for various SWI/SNF subunits and lineage-specific markers (SOX2 and AR) in a patient FFPE sample of mixed PCa with an adenocarcinoma component and a dedifferentiated component. The case illustrates intra-tumor heterogeneity in the expression levels of SWI/SNF subunits. In particular, the dedifferentiated component shows strong SOX2 and BAF45B expression, but lower *SMARCA2* (BRM) expression and a lack AR expression. Scale bars, 50 μm. In total, three cases of mixed PCa from three different patients were tested by IHC (including the case shown here), with concordant results.

Supplementary Figure 6 (related to Fig 1)

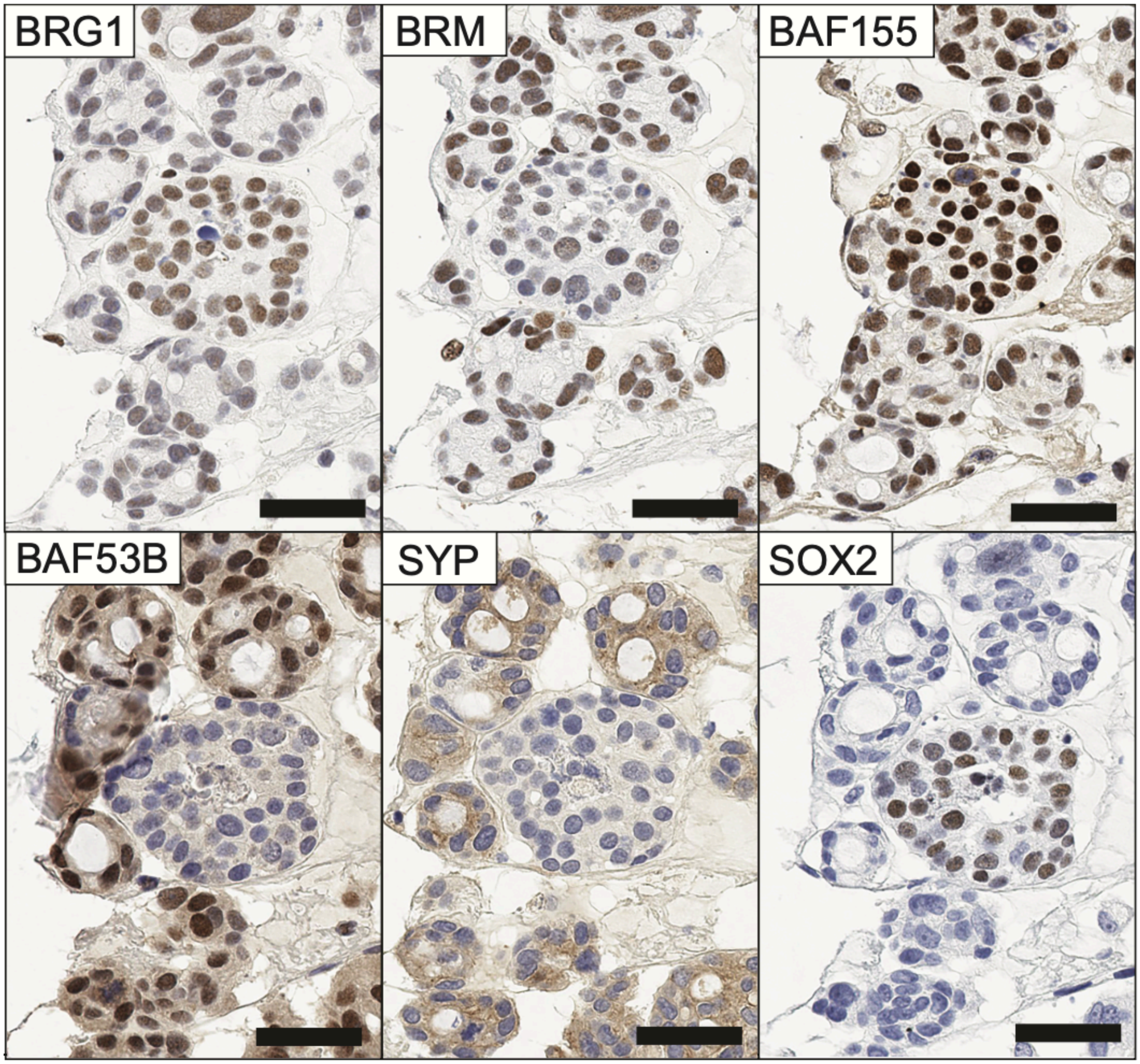

Immunohistochemistry for various SWI/SNF subunits and differentiation markers in a patient tumor-derived CRPC-NE organoid (3D culture) after FFPE processing. The case illustrates intra-tumor heterogeneity in the expression levels of SWI/SNF subunits in organoid cultures. Of note, there is a distinct subpopulation of cells characterized by increased SOX2, BRG1 (*SMARCA4*) and BAF155 expression and low BRM (*SMARCA2*) expression. These clusters could represent a sub-population of less differentiated tumor cells, consistent with increased expression of the "stemness" regulator SOX2 and lacking the expression of terminal neural markers (synaptophysin or BAF53B), although putative tumor-perpetuating properties of this subpopulation remain to be verified functionally. Scale bars, 50 μm. In total, two cases of CRPC-NE organoids (WCM154 and WCM155) from two different patients were tested by IHC (including the case shown here), with concordant results.

Supplementary Figure 7 (related to Fig 1)

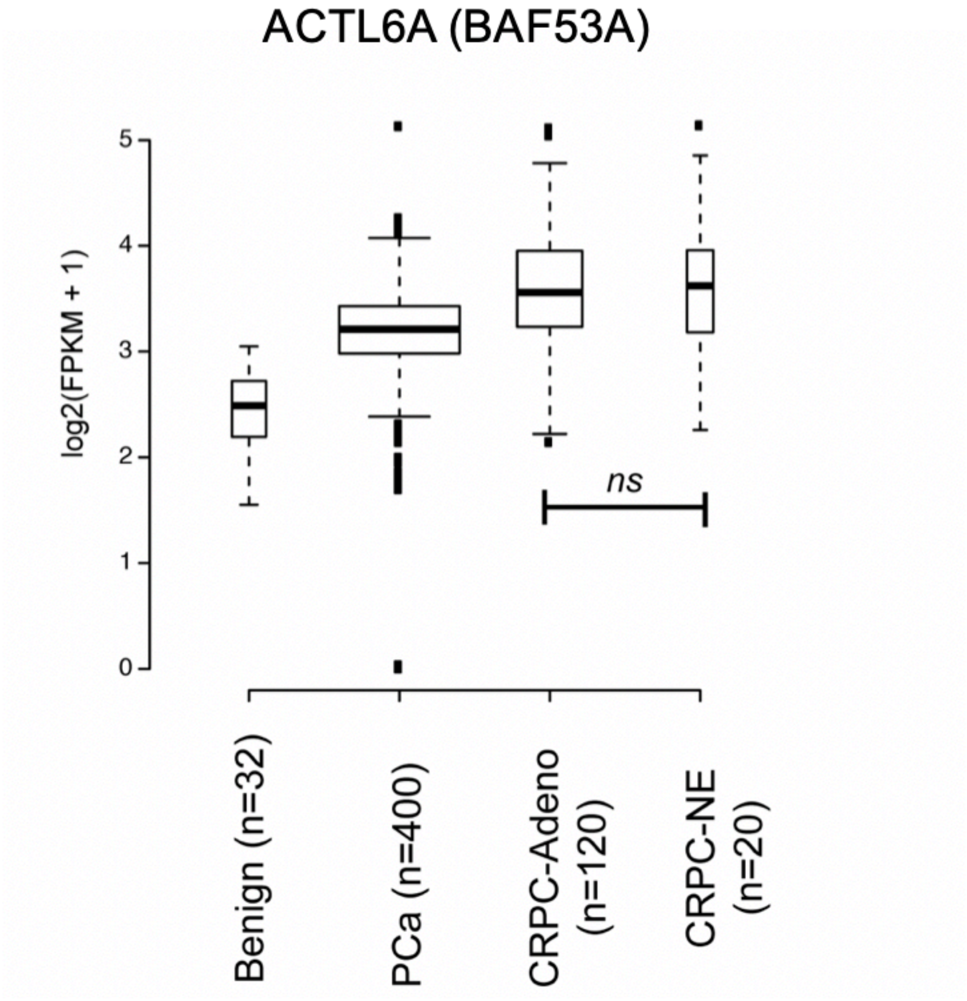

The expression of *ACTL6A* (BAF53A), a paralog of BAF53B, is maintained in CRPC-NE. *ACTL6A* gene expression in patient samples across PCa disease states, showing that *ACTL6A* expression is neither abolished nor decreased in CRPC-NE as compared to CRPC-Adeno (Mann-Whitney Wilcoxon test). The box plots represent the median values and the lower and upper interquartile range (IQR); the upper whisker = min(max(x), Q3 + 1.5 × IQR) and lower whisker = max(min(x), Q1 − 1.5 × IQR), and the outliers are plotted as individual points. Abbreviations: Benign: benign prostatic tissue; PCa: localized, hormone treatment-naïve prostatic adenocarcinoma; CRPC-Adeno: Castration resistant prostate cancer, adenocarcinoma subtype; CRPC-NE: Castration resistant prostate cancer, neuroendocrine subtype.

Supplementary Figure 8 (related to Fig 1)

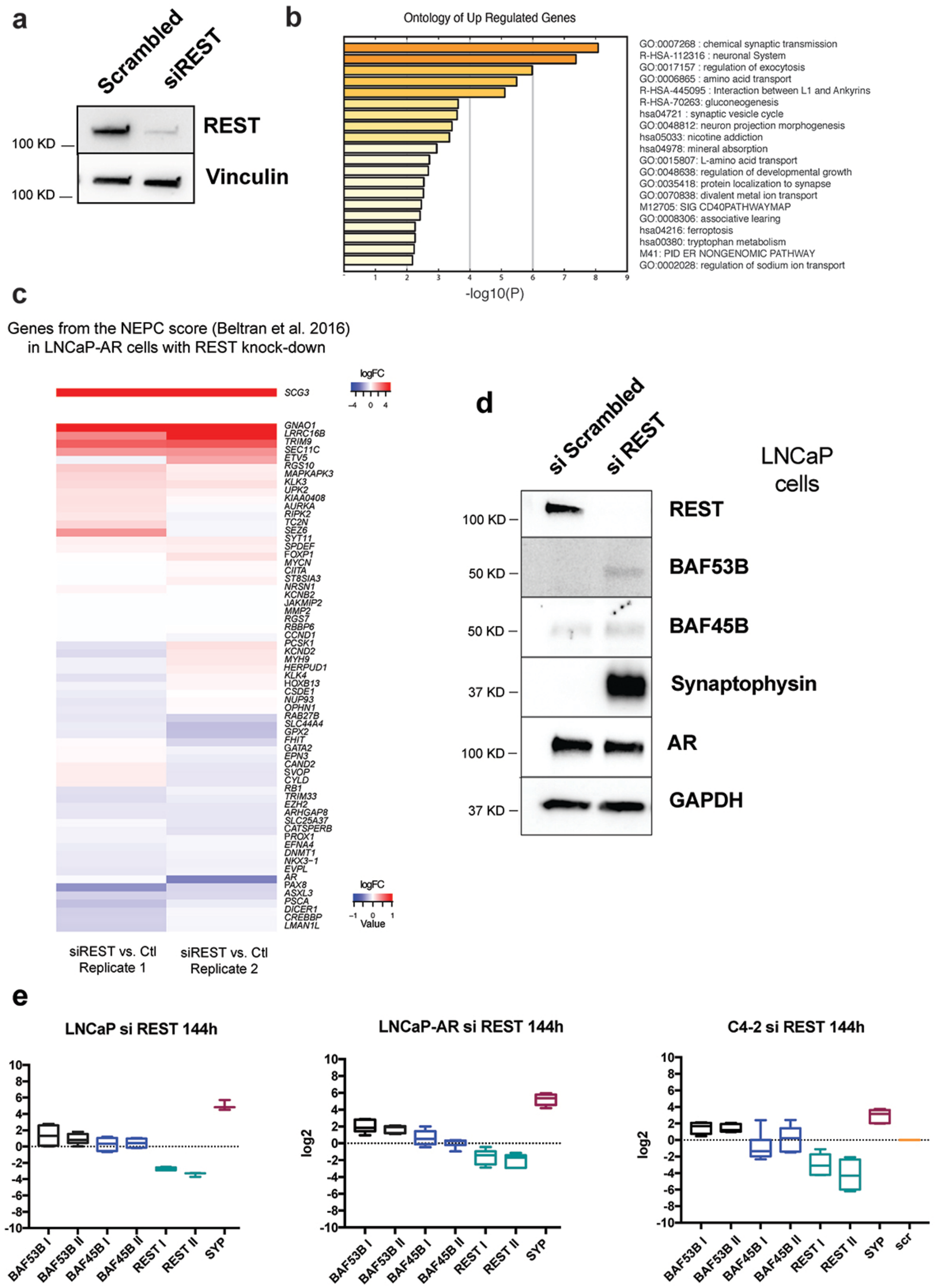

REST knock-down induces expression of neuronal genes (a) immunoblot showing REST knock-down efficiency. **(b)** Ontology of genes upregulated upon REST knock-down based on RNA-seq results, showing a significant upregulation of neuronal gene expression programs (**c**) Transcriptomic changes (assessed by RNA-seq) in prostatic adenocarcinoma cells (LNCaP-AR) upon siRNA-mediated REST knock-down. Heatmap of gene expression levels using genes from

the transcriptomic NEPC score (Beltran et al., 2016); only a few genes from the NEPC signature score, related to terminal neuronal differentiation (e.g., Secretogranin 3, *SCG3*), were upregulated. **(e)** Effects of REST knock-down on the expression of selected genes in prostatic adenocarcinoma cell lines. q-PCR (4 independent replicates) showing strong upregulation of synaptophysin (SYP) mRNA upon REST knock-down, a modest increase in BAF53B mRNA, and no significant change in BAF45B mRNA. I and II indicate different pairs of primers. The box plots represent the median values with upper and lower quartiles. Whiskers are plotted down to the minimum and up to the maximum value. Data were analyzed using one-way ANOVA. SYP increase is significant (p value < 0.0001). Statistical significance was evaluated at 0.05 alpha level with GraphPadPrism, version 8.2.1, Mac. **(d)** Immunoblot showing an induction of synaptophysin expression at the protein level upon REST knock-down in LNCaP cells, a very modest induction of BAF53B expression, and no notable changes in AR or BAF45B expression.

Supplementary Figure 9 (related to Fig 1)

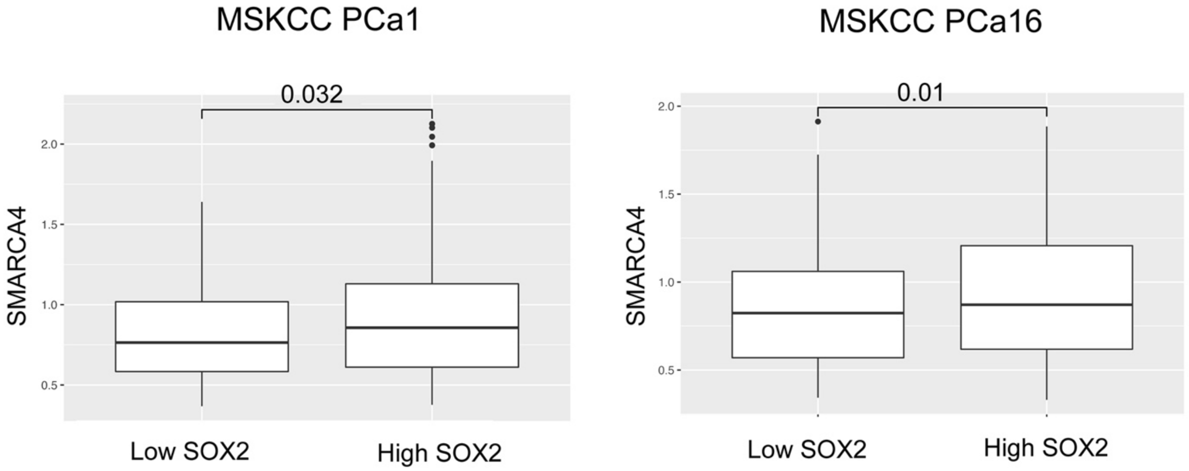

Single cell RNA-seq data from two CRPC-NE organoids in 3D growth, demonstrating significantly higher SMARCA4 expression levels in cells that also show high SOX2 expression (defined as superior to mean SOX2 expression in each experiment); Wilcoxon test. PCa1 N=333, low SOX2 PCa1 N=153, high SOX2 PCa1 N=180, PCa16 N=259, low SOX2 PCa16 N=259, high SOX2 PCa16 N=185. The box plots represent the median values with upper and lower quartiles; the whiskers represent the range outside the quartiles and the outliers are plotted as the individual points. Representation of a two-tailed test.

Supplementary Figure 10 (related to Fig 1)

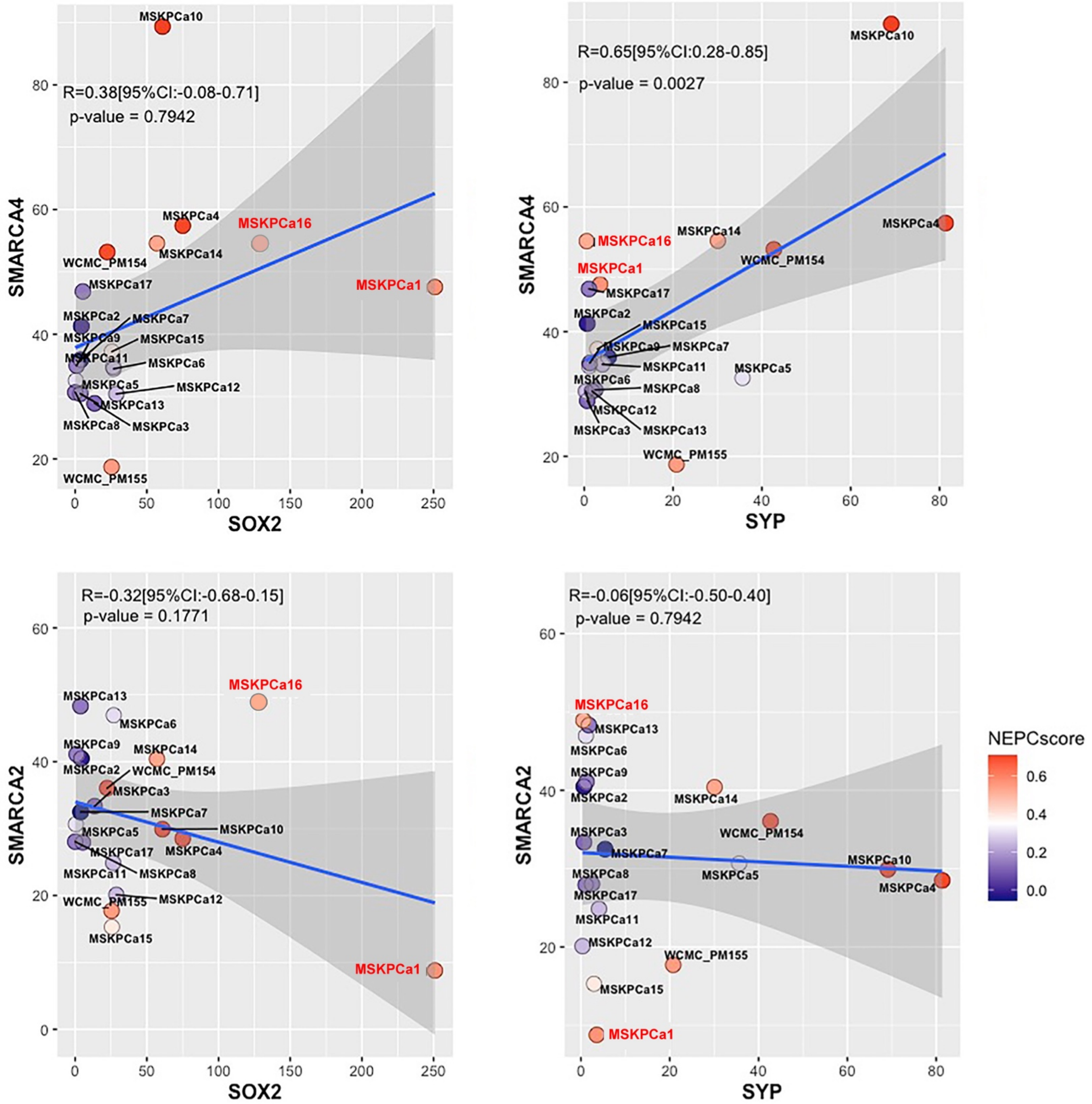

Bulk RNA-seq gene expression values (FPKMs) for selected genes in patient-derived PCa organoids. Pearson correlation analysis between SMARCA4/SMARCA2 and SOX2/Synaptophysin (SYP) is shown in each graph. Organoids are color-coded according to their transcriptomic NEPC score, whereby a score >0.4 indicates a CRPC-NE phenotype and a score<0.4 indicates a CRPC-Adeno phenotype (Beltran et al., PMID 26855148). The two

organoids used in the single cell RNA-seq experiment from Supplementary Fig. S1.9 are indicated in red font.

Supplementary Figure 11 (related to Fig 2)

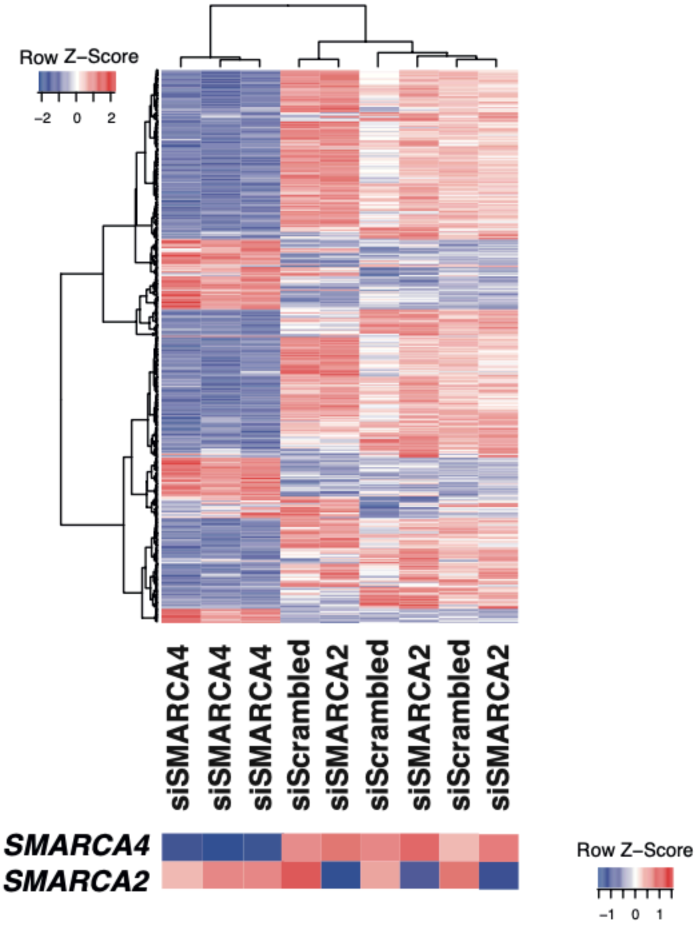

Transcriptomic changes assessed by RNA-seq in LNCaP prostatic adenocarcinoma cells upon *SMARCA4* or *SMARCA2* knock-down. Unsupervised clustering using the 500 most deregulated genes upon *SMARCA4* or *SMARCA2* knock-down. *SMARCA4* depletion has a profound effect on the transcriptome, while the effects of *SMARCA2* depletion are modest. The profiles of top deregulated genes differ between the two knock-down conditions, supporting non-redundant roles of *SMARCA4* and *SMARCA2* in PCa.

Supplementary Figure 12 (related to Fig 2)

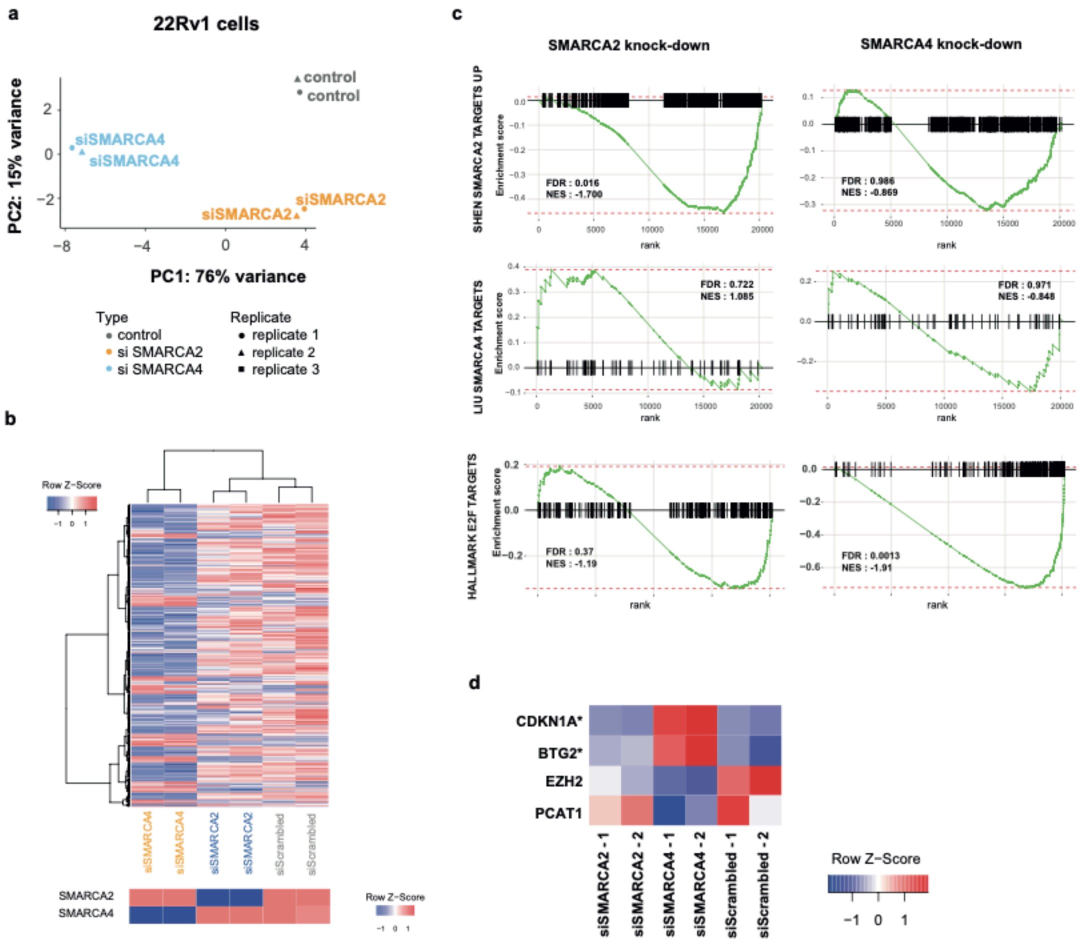

Transcriptomic changes assessed by RNA-seq in 22Rv1 cells line upon *SMARCA4* or *SMARCA2* knock-down. (a) Principal component analysis, showing a marked effect of the *SMARCA4* knock-down on the transcriptome; scrambled siRNA was used as control. **(b)** Unsupervised hierarchical clustering and gene expression heatmap using the 500 most highly deregulated genes, showing that the effects of *SMARCA4* knock-down on the transcriptome are more pronounced that the effects of *SMARCA2* knock-down. **(c)** Gene Set Enrichment Analysis in *SMARCA4* knock-down or *SMARCA2* knock-down to the control. **(d)** Heatmap depicting gene expression levels of selected genes in *SMARCA4* knock-down, *SMARCA2* knock-down and control samples. These four genes were chosen because they were significantly deregulated in LNCaP cells upon *SMARCA4* knock-down. *indicates genes that are also significantly deregulated (FDR<0.05) in 22Rv1 cells upon *SMARCA4* knock-down.

Supplementary Figure 13 (related to Fig 2)

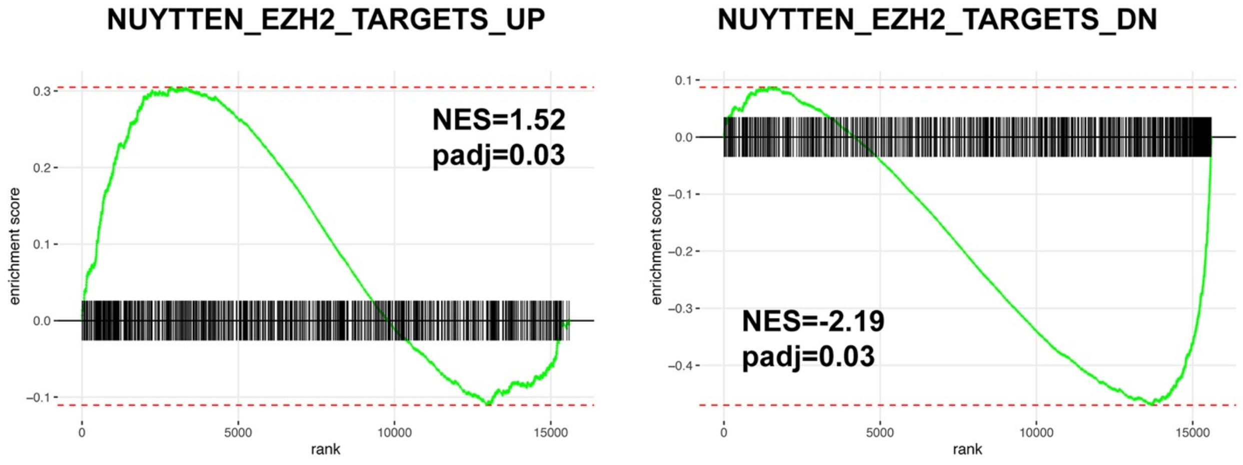

Knock-down of BRG1 and knock-down of EZH2 can produce partly overlapping effects in PCa cells. GSEA in LNCaP cells with BRG1 knock-down (compared to the Scrambled siRNA control). Results for two EZH2-related gene setsare shown - genes upregulated in PC3 prostate cancer cells after knockdown of EZH2 (left) and genes downregulated in PC3 cells after knockdown of EZH2 (right).

Supplementary Figure 14 (related to Fig 2)

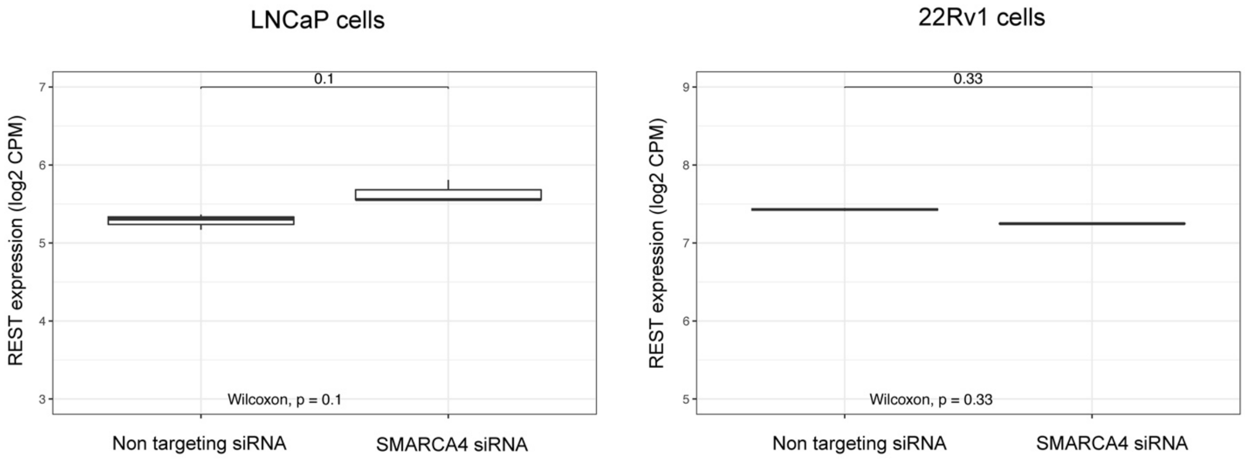

Gene expression levels of the *REST* gene upon *SMARCA4* knock-down, assessed by RNA-seq in LNCaP and 22Rv1 cells. There is no statistically significant difference in *REST* expression between the SMARCA4 knock-down condition and the control (Scrambled siRNA). Wilcoxon test. Representation of a two-tailed test.

Supplementary Figure 15 (related to Fig 2)

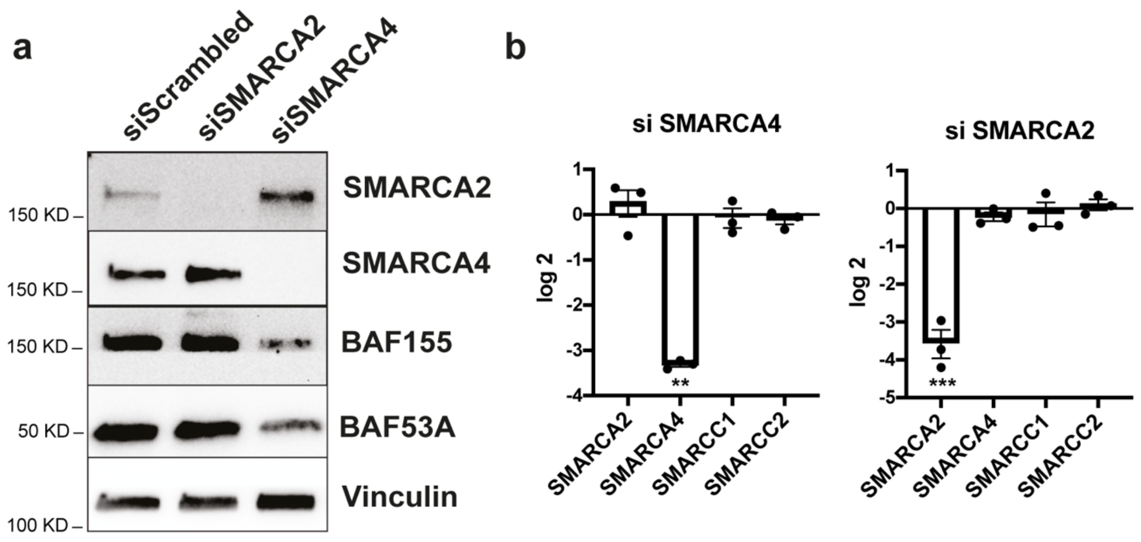

Effects of *SMARCA4* or *SMARCA2* knock-down on expression levels of other SWI/SNF subunits in LNCaP cells. (a) Immunoblot showing that the protein levels of the SWI/SNF subunits BAF155 (*SMARCC1*) and BAF53A decrease upon *SMARCA4* depletion, but not upon *SMARCA2* depletion. **(b)** q-PCR showing that changes of BAF155 (*SMARCC1*) expression at the protein level are not explained by changes at the mRNA level, suggesting that the decreased protein levels may be due to destabilization of the complex and degradation of the released subunits, rather than a decrease in transcription. N= 3 independent experiments. Data are presented as mean values +/- SEM and were analyzed using one-way ANOVA (** p=0.0012 and ****p* =0.0004). Adjustments were made for multiple comparisons. Statistical significance was evaluated at 0.05 alpha level with GraphPadPrism, version 8.2.1, Mac. Source data are provided as a Source Data file.

Supplementary Figure 16 (related to Fig 2)

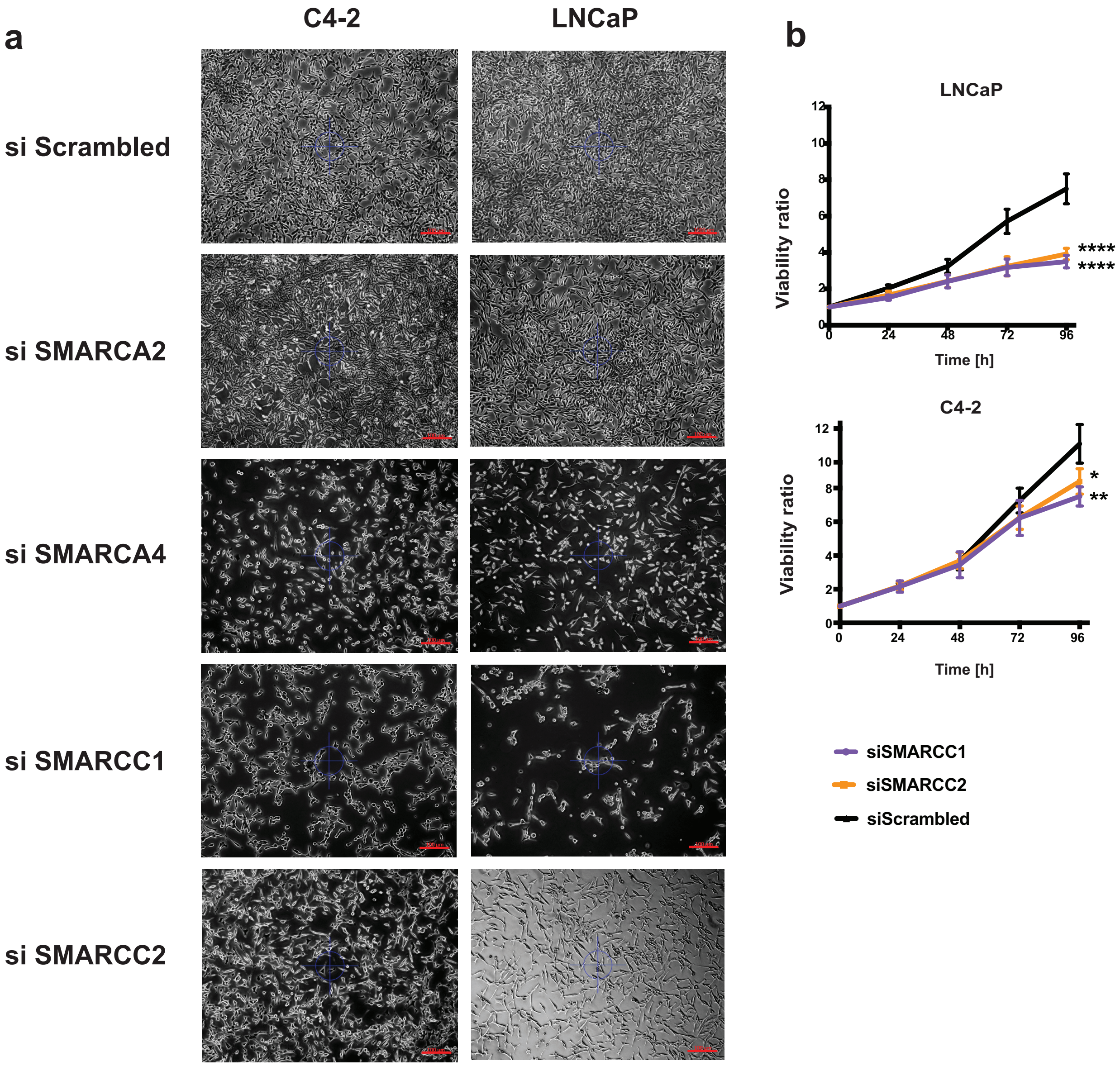

Knock-down of *SMARCA4*, *SMARCC1* and *SMARCC2* impacts cell growth in PCa cells. (a) Brightfield microcopy showing the effects of siRNA-mediated knock-down of SWI/SNF subunits on PCa cell growth *in vitro*. C4-2 cells (CRPC-Adeno) and LNCaP cells (prostatic adenocarcinoma) 96h after siRNA treatment against *SMARCA2, SMARCA4, SMARCC1* or *SMARCC2.* Cells treated with Scrambled siRNA are shown as control. Scale bars (red): 100 μm. **(b)** Effects of BAF155 (*SMARCC1*) or BAF170 (*SMARCC2*) knock-down on PCa cell growth. LNCaP: adenocarcinoma cells, C4-2: CRPC-Adeno cells. Two-way ANOVA test (*p=0.0216, **p=0.0056, ****p<0.0001). The curves represent pooled results from 3 replicate experiments (bars, standard error).

Supplementary Figure 17 (related to Fig 2)

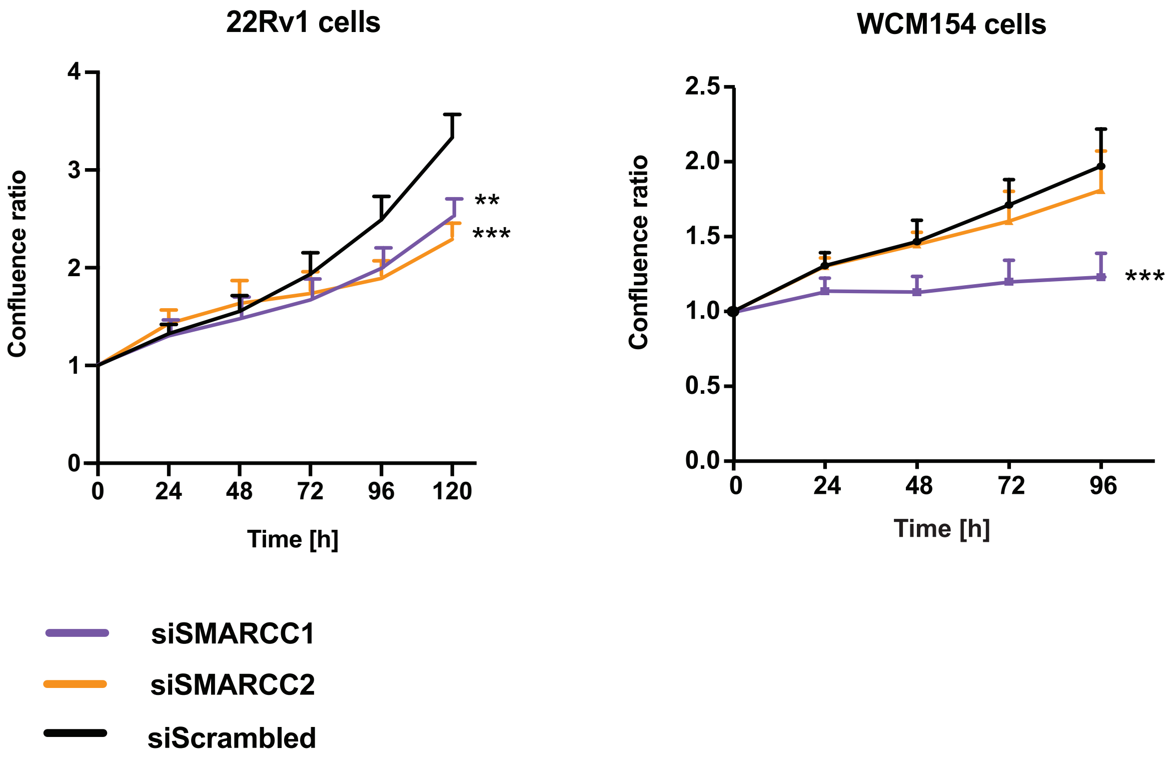

Knock-down of *SMARCC1* and *SMARCC2* impacts cell growth in PTEN-competent PCa cells lines. The experiments show that PCa cells, including the CRPC-NE cell line WCM154, are sensitive to knock-down of the SWI/SNF subunit *SMARCC1* (BAF155). The curves represent pooled results from 3 independent experiments (bars, standard error). 22Rv1: prostatic adenocarcinoma cell line, WCM154: CRPC-NE patient tumor-derived organoid cells in 2D culture. Two-way ANOVA test (22Rv1: **p=0.027 and ***p=0.002, WCM154: ***p=0.0009). The curves represent pooled results from 3 (22Rv1) and 4 (WCM154) biologically independent experiments. Data are presented as mean values +/- SEM.

Supplementary Figure 18 (related to Fig 2)

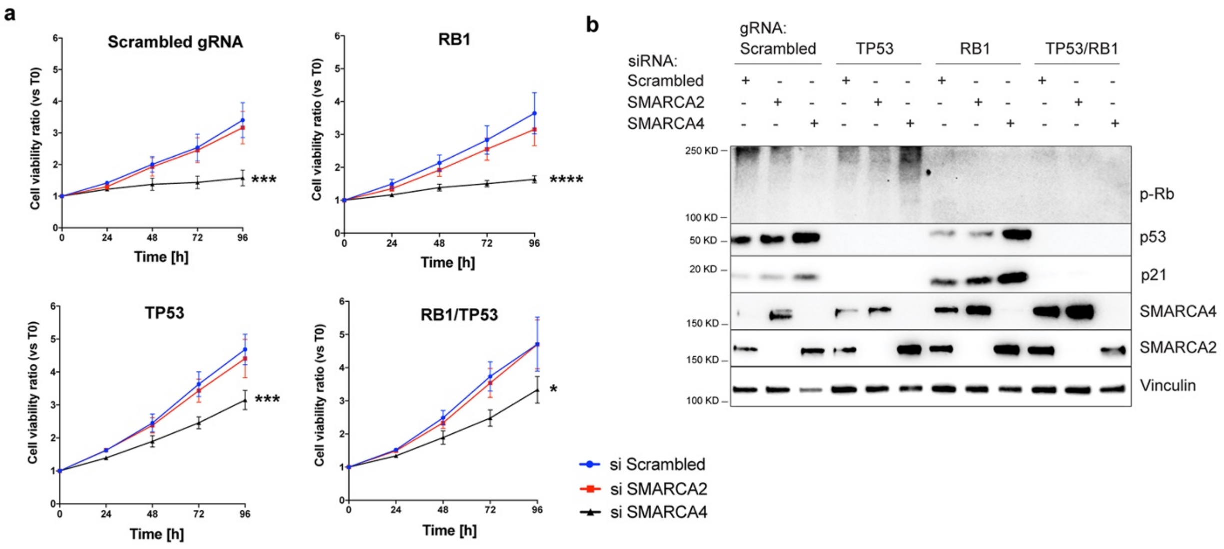

The effects of *SMARCA4* knock-down on cell growth in LNCaP cells are not entirely abrogated by p53 or Rb loss. **(a)** Cell growth curves in control cells (Scrambled gRNA), RB1-negative cells, p53-negative cells or double Rb1/p53 negative cells upon *SMARCA4* knock-down, *SMARCA2* knock-down or treatment with Scrambled siRNA; n=3 biologically independent experiments. Data are presented as mean values +/- SEM and analyzed using two-way ANOVA test. Adjustments were made for multiple comparisons. *p<0.05, **p<0.001, ****p<0.0001 (for Scrambled gRNA: ***p=0.0003, for RB1: ****p<0.0001, for TP53: ***p=0.0007, for RB1/TP53: *p=0.021). **(b)** Immunoblot validation of CRISPR-Cas9 mediated loss of p53 and/or Rb and of siRNA-mediated knock-down of *SMARCA4* (BRG1) or *SMARCA2* (BRM). Source data are provided as a Source Data file.

Supplementary Figure 19 (related to Fig 2)

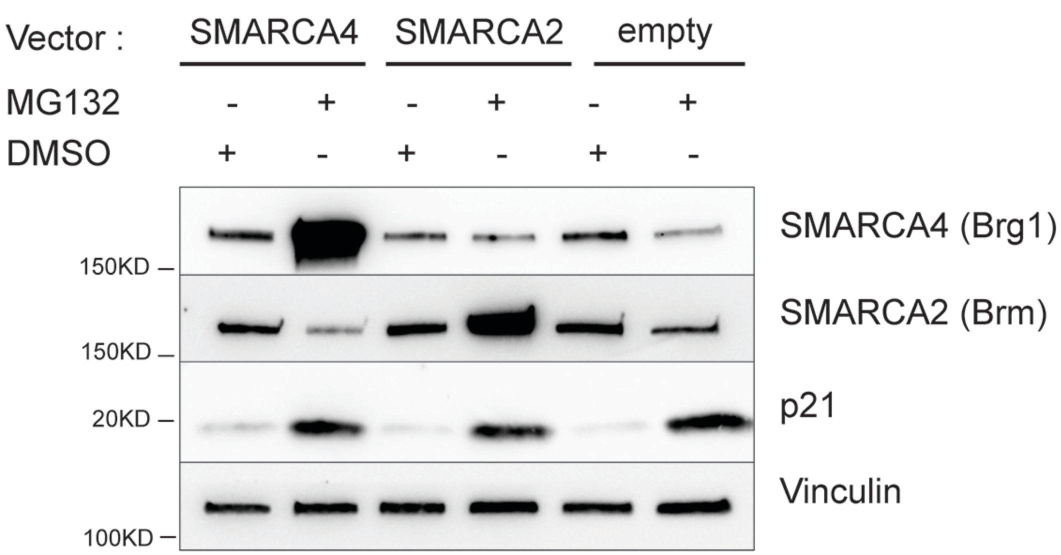

Immunoblot showing BRG1 (SMARCA4) and BRM (SMARCA2) expression levels in 22Rv1 cells transduced with lentiviral vectors and treated for 24h with MG-132 or DMSO (control). The expression levels of the p21 protein are shown as control for the MG-132 treatment. N=1 (one pilot experiment assessing feasibility). Samples shown in the Brg1 and Brm rows derive from the same experiment, but the images come from two separate blots, which were ran and processed in parallel. Equilibrated loading in both blots was confirmed using the loading control marker (vinculin) on each blot. Source data are provided as a Source Data file.

Supplementary Figure 20 (related to Fig 2)

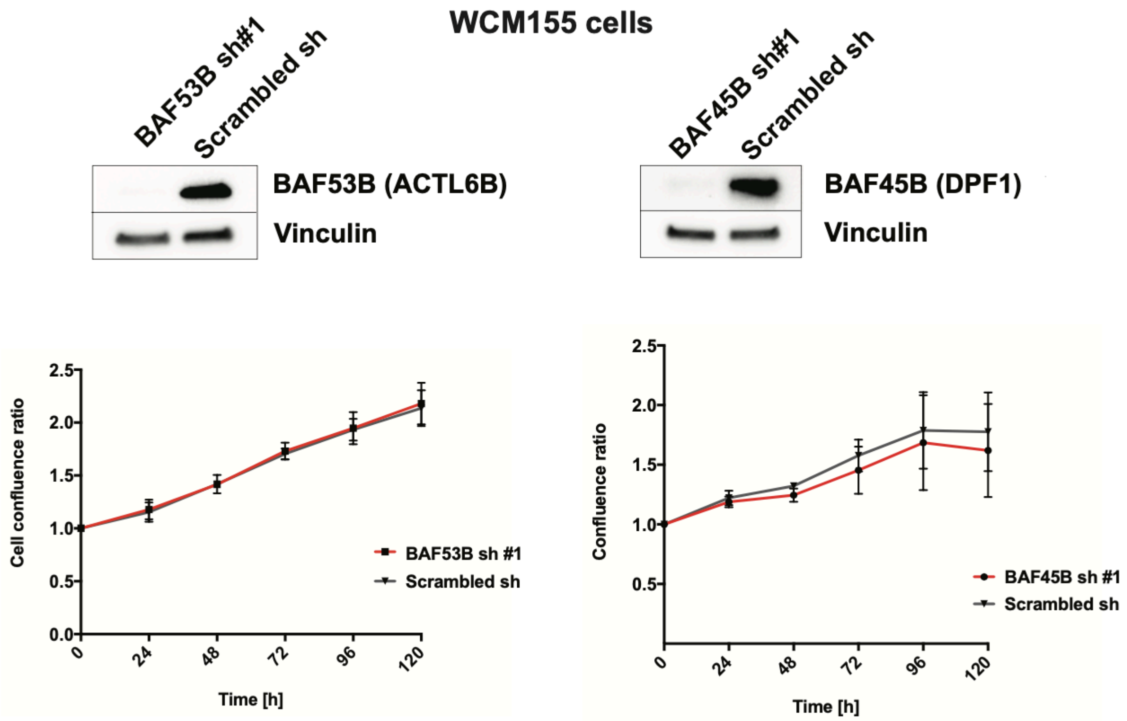

The effects of BAF53B or BAF45B shRNA-mediated knock-down on cell growth of a CRPC-NE patient tumor organoid-derived 2D cell line (WCM155). The immunoblots show knock-down efficiency control (one representative experiment). Each growth curve shows pooled results from three independent experiments (bars: standard error). Source data are provided as a Source Data file.

Supplementary Figure 21 (related to Fig 3)

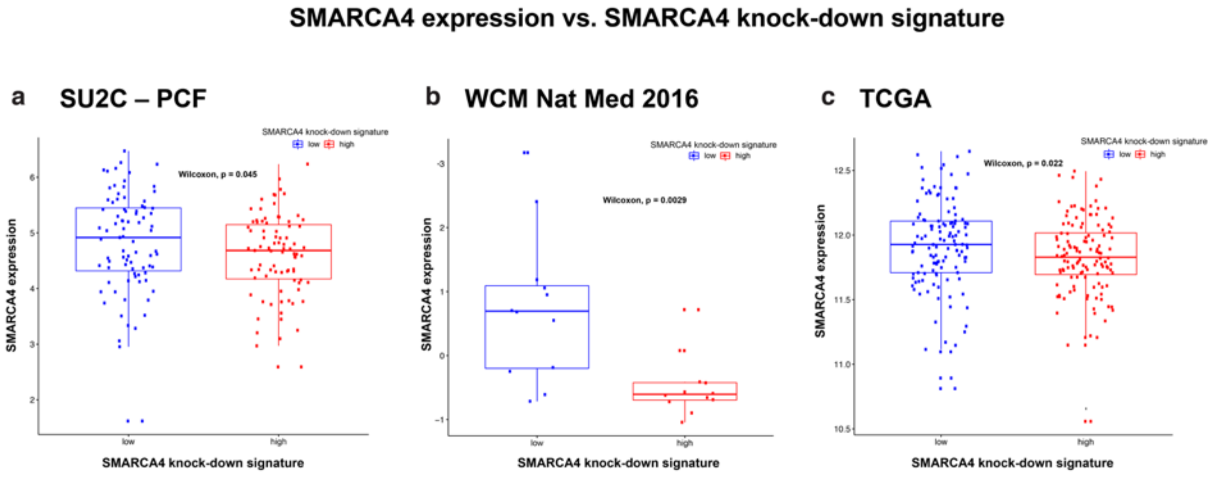

Box plots comparing *SMARCA4* mRNA levels and SMARCA4 knock-down signature score values across three PCa patient cohorts. Each dot represents a sample. *SMARCA4* mRNA expression levels are consistent with the predicted signature score (samples with lower *SMARCA4* expression show higher *SMARCA4* knock-down signature scores, and *vice versa*). Mann-Whitney Wilcoxon test. TCGA N= 495 samples, of which 124 were classified SMARCA4 high. SU2C N= 332 samples, of which 83 were classified SMARCA4 low and 83 cases as SMARCA4 high. WCMN=49 samples, of which 12 were classified as SMARCA4 low and 12 as SMARCA4 high. The box plots represent the median values with upper and lower quartiles; the whiskers represent the range outside the quartiles and the outliers are plotted as the individual points.

Supplementary Figure 22 (related to Fig 3)

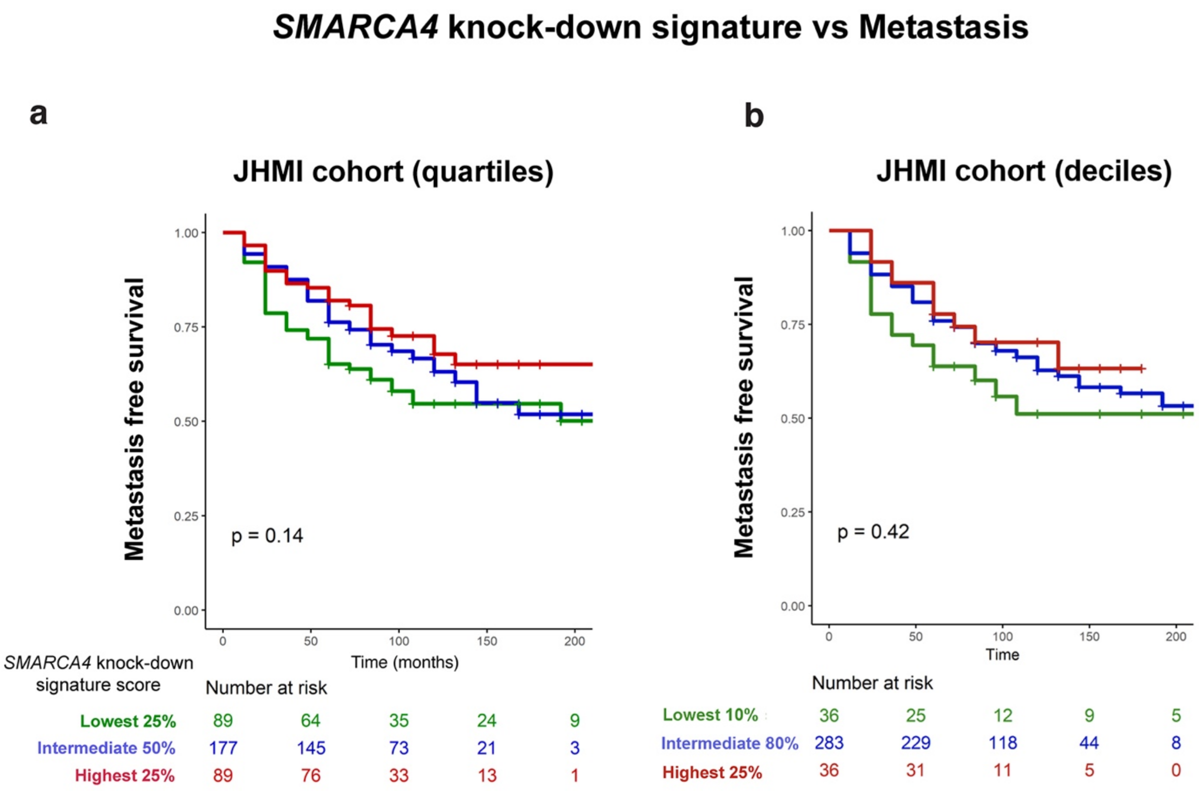

***SMARCA4* knock-down signature vs Metastasis in the JHMI cohort**. Patients were stratified based on quartiles **(a)** or on deciles **(b)** of *SMARCA4* knock-down signature scores, to test for associations between *SMARCA4* knock-down signature scores and metastasis-free survival. There is a trend of lower *SMARCA4* knock-down signature scores being associated with worse metastasis-free survival, but it did not reach statistical significance. Kaplan-Meier analysis and Cox proportional hazard model used; p values shown in **(a)** and **(b)** pertain to statistical analysis comparing groups with lowest (green) and highest (red) *SMARCA4* knock-down signature scores.

Supplementary Figure 23 (related to Fig 3)

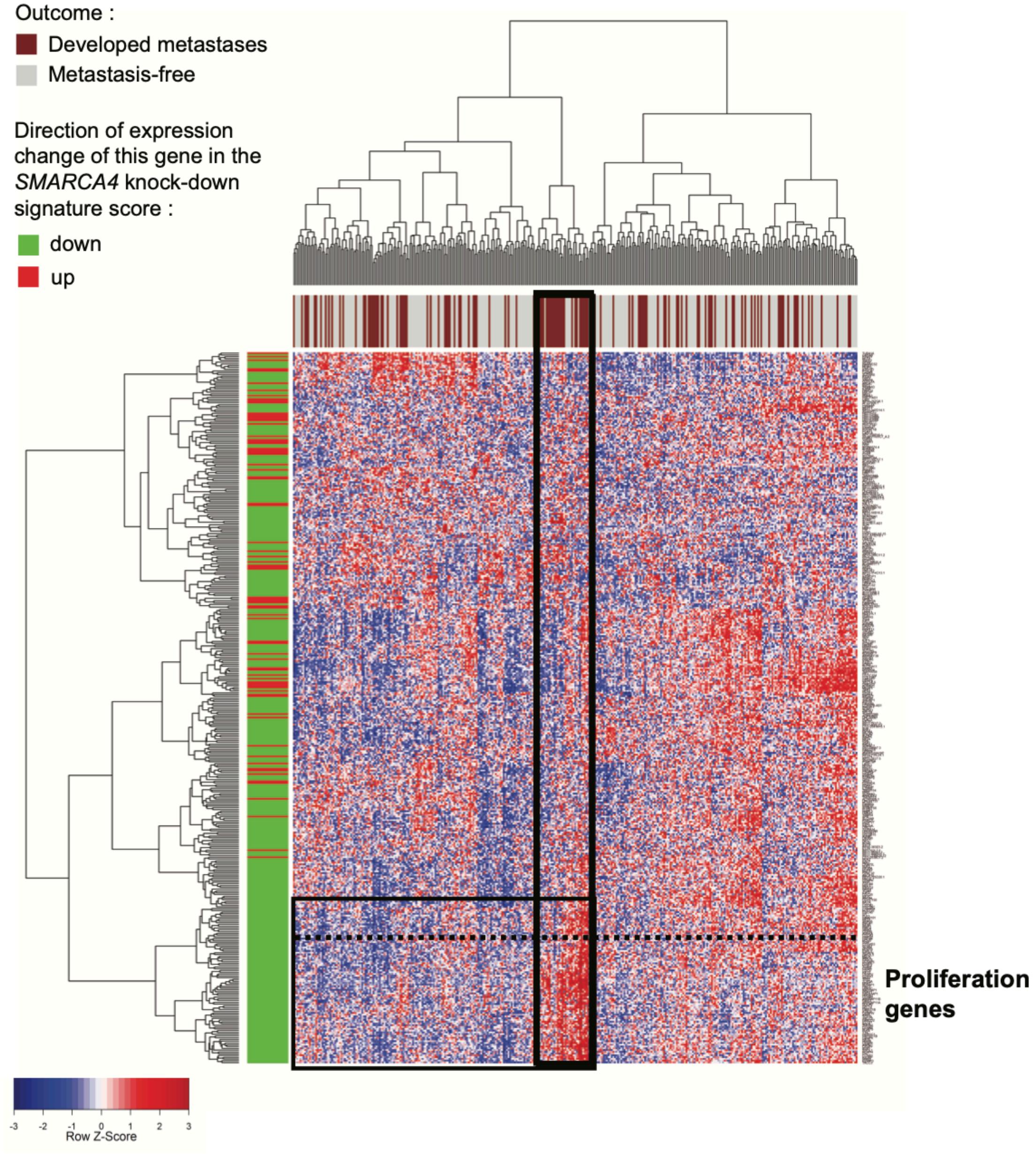

Heatmap of *SMARCA4* knock-down signature genes in the JHMI natural history PCa cohort (Johns Hopkins Medical Institute, n=355) with respect to metastatic outcome. Overexpression of a subset of genes from the signature is seen in a cluster of patients who presented metastatic outcome (black box).

Supplementary Figure 24 (related to Fig 4)

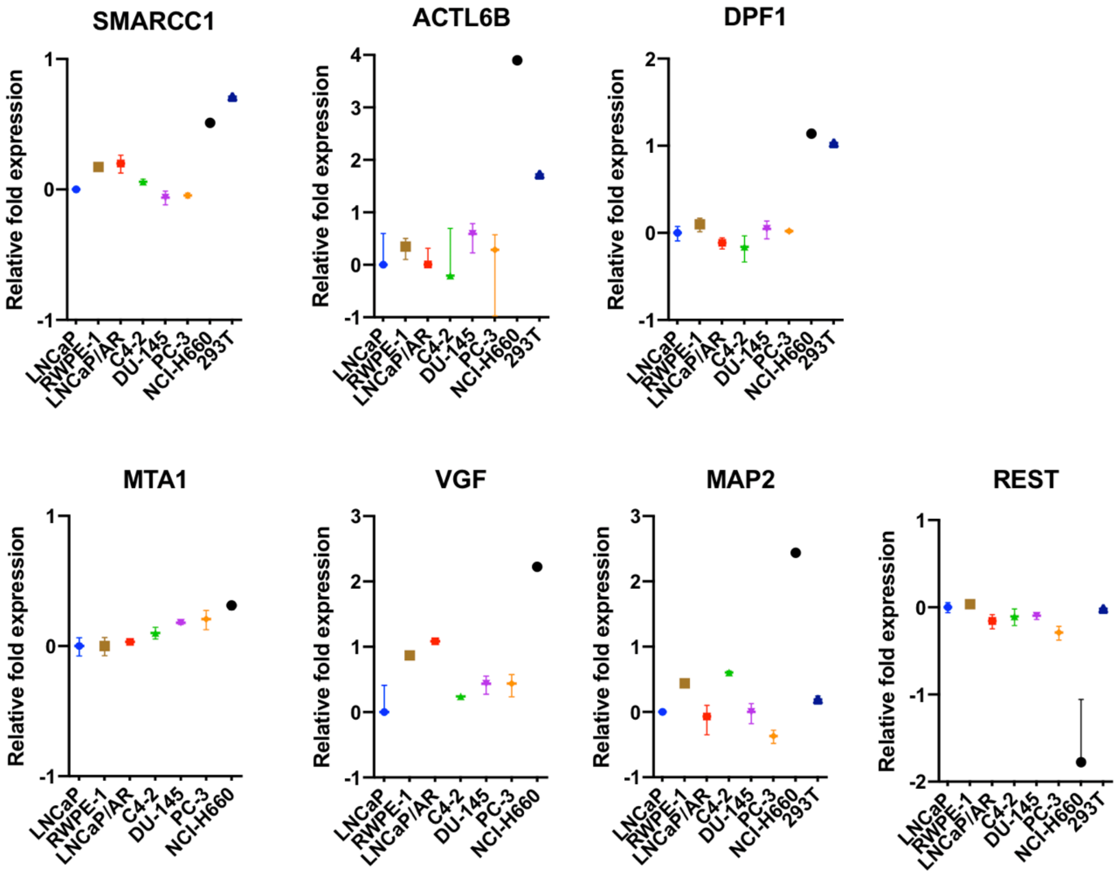

Genes encoding factors that show differential binding to SWI/SNF between CRPC-NE and prostate adenocarcinoma cell lines are also differentially expressed at the transcript level across prostate cancer cell lines. Graphs show gene expression levels assessed by RT-PCR. Relative mRNA levels of each gene shown were normalized to the expression of the average of housekeeping genes *GAPDH* and *ACTB*. Each graph represents three replicates and bars show standard deviation (SD).

Supplementary Figure 25 (related to Fig 4)

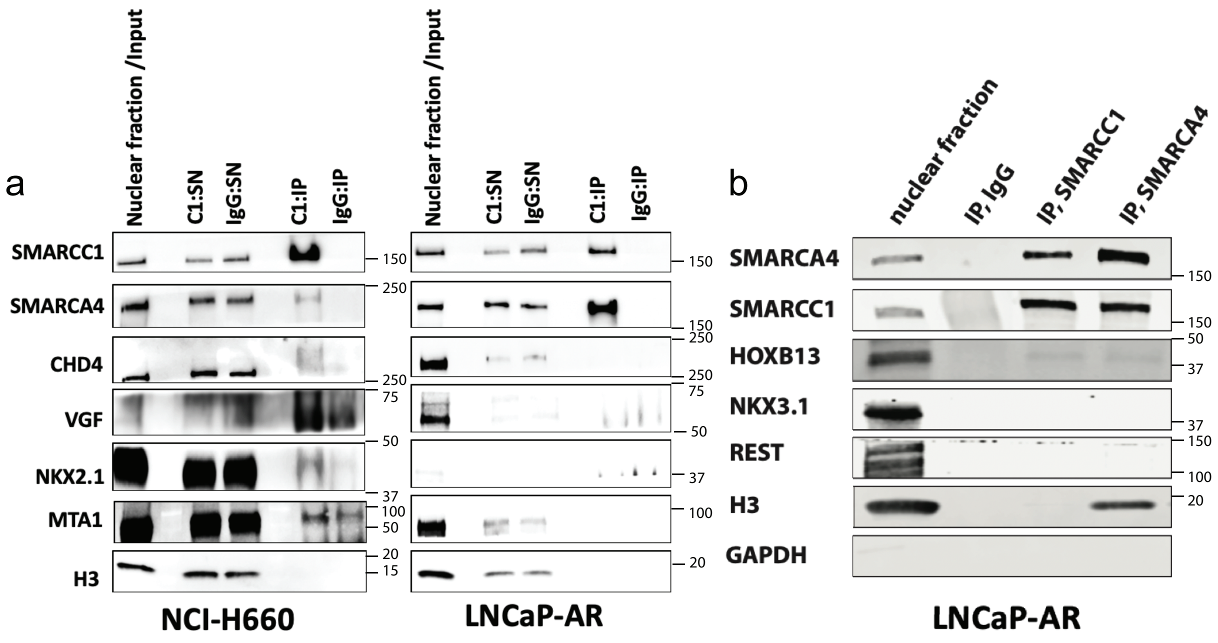

Confirmation of factors associating with BAF155 (SMARCC1) in NCI-H660 and in LNCaP-AR cells by co-immunoprecipitation (co-IP) and immunoblotting. (a) Co-IP of BAF155 (SMARCC1) followed by immunoblotting for BRG1 (SMARCA4), CHD4, VGF, NKX2.1 (TTF1), MTA1 and H3 in nuclear extracts from the CRPC-Adeno cell line LNCaP-AR and from the CRPC-NE cell line NCI-H660. In NCI-H660, MTA1, NKX2.1, VGF and CHD4 were pulled down in the SMARCC1 Co-IP condition, but were either absent or only weakly expressed in the IgG Co-IP (in accordance with mass spectrometry findings). In LNCaP-AR, most of these factors were detectable in the nuclear fraction, but were not detected in the SMARCC1 Co-IP. SMARCA4 (positive control) was pulled down in the Co-IP experiment for both cell lines. C1:IP indicates SMARCC1 Co-IP; IgG indicates control Co-IP with isotype antibody; SN indicates supernatant and serves as control to indicate how much protein was pulled down; H3 serves as nuclear fraction control. (b) Nuclear extract Co-IP of SMARCC1 and of SMARCA4 followed by immunoblotting for SMARCA4, SMARCC1, HOXB13, NKX3.1, REST and H3 in the CRPC-Adeno cell line LNCaP-AR. The immunoblot confirms that HOXB13 co-immunoprecipitated with SMARCC1 and SMARCA4 (in accordance with mass spectrometry findings). Co-immunoprecipitation of NKX3.1 and REST with SMARCA4 or SMARCC1 could not be confirmed in this immunoblot. Source data are provided as a Source Data file.

Supplementary Figure 26 (related to Fig 4)

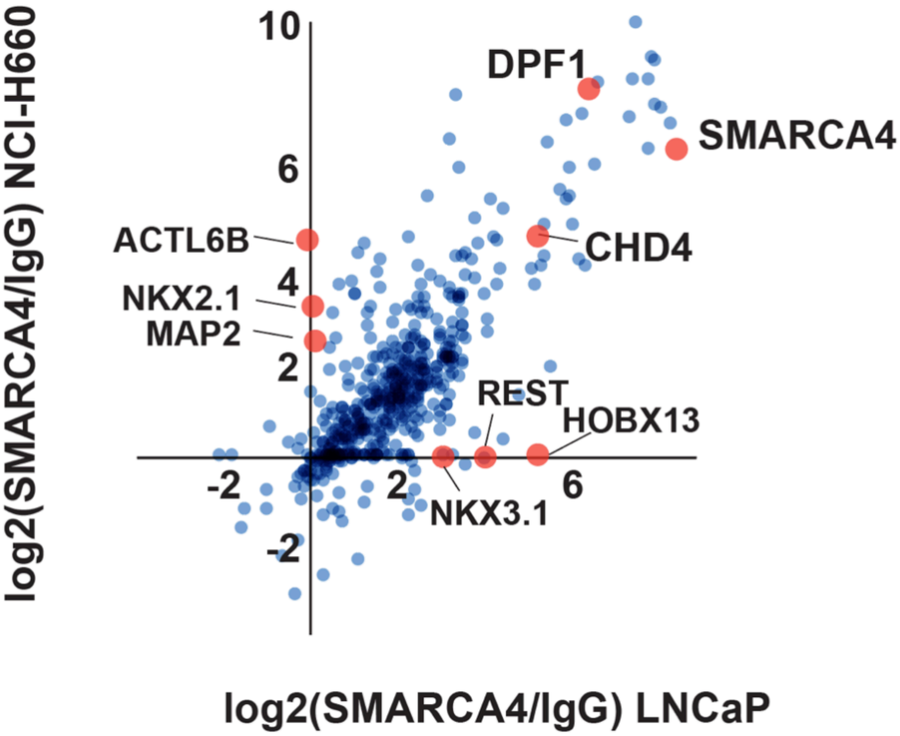

SWI/SNF associates with different transcriptional regulators in CRPC-NE and in adenocarcinoma cells. A qualitative representation comparing proteins associated with SWI/SNF in NCI-H660 (CRPC-NE) and in LNCaP (adenocarcinoma) cells (averaged data from two co-IP experiments). Plotted are log2 fold change values of SMARCA4/IgG in NCI-H660 (y-axis) versus LNCaP (x-axis), for proteins present in both cell lines with sufficient evidence in each cell line (i.e. if present in two replicates of at least one condition).

Supplementary Figure 27 (related to Fig 4)

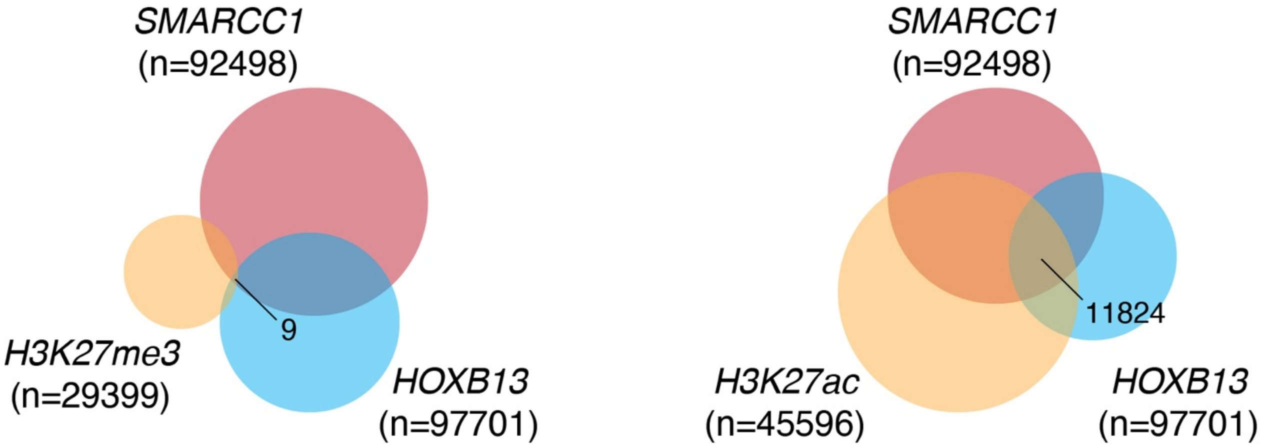

SWI/SNF colocalizes with HOXB13 at active chromatin sites in prostatic adenocarcinoma cells. Venn diagrams illustrating the overlap in genome occupancy sites for SMARCC1, HOXB13, H3K27me3 and H3K27ac in LNCaP cells, assessed by ChIP-seq (analysis performed using published datasets).

Supplementary Figure 28 (related to Discussion)

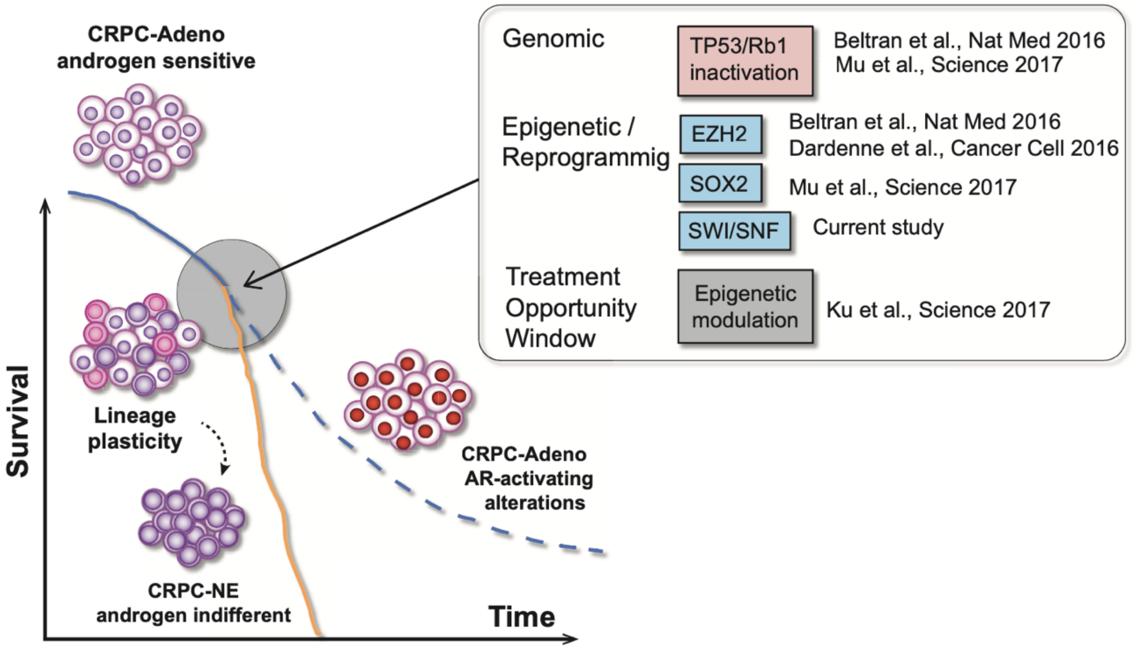

Lineage plasticity as a mechanism of disease progression in CRPC. Schematic representation of the current state of knowledge and potential place of SWI/SNF in this mechanism. Abbreviations: CRPC-Adeno: Castration resistant prostate cancer, adenocarcinoma subtype, CRPC-NE: Castration resistant prostate cancer, neuroendocrine subtype, AR: Androgen receptor.

Supplementary tables

Supplementary Table 1 (Related to Fig 2). Analysis of association between *SMARCA4* (BRG1) and *SMARCA2* (BRM) protein expression (strong vs. moderate/weak/negative) and patient's overall survival adjusted for single covariates (factors with known impact on PCa prognosis). Cox proportional hazards models were executed (univariable and multivariable), all p-values were two-sided with statistical significance evaluated at the 0.05 alpha level. Adjustments were not made for multiple comparisons due to the exploratory nature of the analyses.

	HR	95% CI	p.value
BRG1_cat2Strong	2.17	1.07, 4.42	0.03

	HR	95% CI	p.value
BRM_cat2Strong	0.5	0.24, 1.04	0.06

	HR	95% CI	p.value
BRG1_cat2Strong	2.3	1.13, 4.69	0.02
Positive.LN1	0.58	0.26, 1.26	0.17

	HR	95% CI	p.value
BRG1_cat2Strong	2.2	1.07, 4.53	0.03
Gleason.grade.group 1	2.21	0.29, 16.89	0.45
Gleason.grade.group 2/3	4.11	0.53, 32.08	0.18
Gleason.grade.group 4/5	2.05	0.22, 18.73	0.52

	HR	95% CI	p.value
BRG1_cat2Strong	2.12	1.03, 4.36	0.04
Extraprostatic.extension1	1.24	0.42, 3.61	0.7

	HR	95% CI	p.value
BRG1_cat2Strong	2.21	1.08, 4.51	0.03
Positive margins	0.79	0.36, 1.73	0.55
Margins: data not available	0.88	0.33, 2.35	0.79

	HR	95% CI	p.value
BRG1_cat2Strong	2.14	1.05, 4.35	0.04
ADJ_AB1	1.87	0.65, 5.35	0.25

	HR	95% CI	p.value
BRG1_cat2Strong	2.13	1.04, 4.34	0.04
ADJ_RADI1	1.57	0.47, 5.21	0.46

	HR	95% CI	p.value
BRM_cat2Strong	0.46	0.22, 0.97	0.04
Positive.LN1	0.57	0.26, 1.25	0.16

	HR	95% CI	p.value
BRM_cat2Strong 0.51	0.51	0.24, 1.07	0.08
Gleason.grade.group 1	2.48	0.33, 18.88	0.38
Gleason.grade.group 2/3	4.24	0.54, 33.07	0.17
Gleason.grade.group 4/5	2.25	0.25, 20.39	0.47

	HR	95% CI	p.value
BRM_cat2Strong	0.5	0.24, 1.04	0.06
Extraprostatic.extension1	1.44	0.5, 4.16	0.5

	HR	95% CI	p.value
BRM_cat2Strong	0.49	0.23, 1.04	0.06
Positive margins	0.86	0.39, 1.9	0.71
Margins: data not available	1.05	0.39, 2.81	0.93

	HR	95% CI	p.value
BRM_cat2Strong	0.48	0.23, 1.01	0.05
ADJ_AB1	2.02	0.7, 5.8	0.19

	HR	95% CI	p.value
BRM_cat2Strong	0.47	0.22, 0.99	0.05
ADJ_RADI1	2.08	0.62, 7	0.23

Supplementary Table 2 (Related to Fig 3). Fisher's statistical test comparing the NEPC transcriptomic score or the AR signaling score and *SMARCA4* knock-down signatures score, using cases from the SU2C-PCF and WCM Nat Med 2016 cohorts. Representation of a two-tailed test.

**Cohort**			**SMARCA4 knock-down signature**		**Fisher test**
			Low	High	**p value**
SU2C-PCF	NEPC score	≥ 0.4	19	1	1.40E-05
		< 0.4	63	79	
	AR signaling	≤ 0.25	27	7	0.0001
		> 0.25	55	73	
WCM	NEPC score	≥ 0.4	7	1	0.009
		< 0.4	5	11	
	AR signaling	≤ 0.25	8	2	0.03
		> 0.25	4	10	

Supplementary Table 3 (Related to Methods). List of antibodies, dilutions and experimental conditions used in immunohistochemistry and in immunoblotting experiments

Protein name	Antibody information			Immunoblotting	Immunohistochemistry		
	Company	Clone name	Catalogue number	Dilution	Dilution	Retrieval solution (pH)	Retrieval time
AR	Abcam	ER179(2)	ab108341	1/10000	-	-	-
BAF155	Abcam	EPR12395	ab172638	1/5000	1/300	H1 (pH6)	30 min
BAF170	Cell Signaling Technology	D8O9V	12760	1/10000	1/300	H1 (pH6)	30 min
BAF45B	Atlas Antibodies	polyclonal	HPA049148	1/1000	1/100 with casein	H2 (pH9)	40 min
BAF47 (INI-1)	BD Biosciences		bd612110	-	1/100	H2 (pH9)	30 min
BAF47 (INI-1)	Abcam	EPR12014	ab181976	1/5000	-	-	-
BAF53A	Abcam	EPR7443	ab131272	1/2000	-	-	-
BAF53B	Abcam	EP10101	ab180927	1/1000	1/50	H2 (pH9)	20 min
Brg1	Abcam	EPR3912	ab108318	1/1000	1/50	H2 (pH9)	60 min
Brm	Cell Signaling Technology	D9E8B	11966	1/1000	1/200	H1 (pH6)	30 min
CHD4	Cell Signaling Technology	D8B12	11912	1/1000			
EZH2	Active Motif	polyclonal	39933	1/5000	-	-	-
GAPDH	Millipore Sigma	polyclonal	AB2302	1/10000	-	-	-
Histone H3	Cell Signaling Technology	D1H2	4499	1/1000			
HOXB13	Novus Biologicals	polyclonal	NBP2-48778	1/500			
Ki-67	Dako	MIB-1	M7240	-	1/50	H2 (pH9)	20 min
MAP2	Abcam	polyclonal	ab32454	1/500			
Mouse Anti-rabbit IgG (Conformation Specific)	Cell Signaling Technology	L27A9	5127	1/2000			
MTA1	Cell Signaling Technology	D40DY	5647	1/1000	-	-	-
NKX3.1	Cell Signaling Technology	D2Y1A	83700	1/1000	-	-	-
p-Rb1	Cell Signaling Technology	D20B12	8516	1/1000	-	-	-
p21	Cell Signaling Technology	12D1	2947	1/1000	-	-	-
p53	Santa Cruz	DO-1	sc-126	1/1000	-	-	-
Rb1	Abcam				-	-	-
REST	Millipore Sigma	polyclonal	07-579	1/1000	-	-	-

SOX2	Cell Signaling Technology	D6D9	3579	1/1000	1/100	H2 (pH9)	20 min
synaptophysin	Thermo Scientific	SP11	RM9111-S	-	1/100	H2 (pH9)	20 min
synaptophysin	Abcam	YE269	ab32127	1/1000	-	-	-
TTF1 / NKX2.1	Abcam	EP1584Y	ab76013	1/2000	-	-	-
VGF	Abcam	polyclonal	ab69989	1/500			
Vinculin	Abcam	EPR8185	ab129002	1/5000	-	-	-

Supplementary Table 4 (Related to Methods). Prostate cancer cell lines and organoids used in this study

	Name	Publication	Subtype
**Cell lines**	RWPE	Bello et al., 1997, PMID: 9214605	Adeno
	LNCaP	Gibas Z, et al., 1984, PMID: 6584201	Adeno
	LNCaP-AR	Chen et al., 2004, PMID: 14702632	Adeno
	C4-2	Thalmann GN, et al., 1994 PMID: 8168083	Adeno
	DU-145	Papsidero LD, et al., 1981, PMID: 6935463	Adeno
	PC3	Kaighn ME, et al., 1979, PMID: 447482	Adeno
	22RV1	Sramkoski RM, et al., 1999, PMID: 10462204	intermediate
	NCI H660	Lai SL, et al., 1995, PMID: 7762988	NEPC
	EF1		NEPC
			based on NEPC score
**Organoids**	MSKPCa1a	Gao et al., 2014, PMID: 25201530	NEPC
	MSKPCa2	Gao et al., 2014, PMID: 25201530	Adeno
	MSKPCa3	Gao et al., 2014, PMID: 25201530	Adeno
	MSKPCa4	Gao et al., 2014, PMID: 25201530	NEPC
	MSKPCa5	Gao et al., 2014, PMID: 25201530	Adeno
	MSKPCa6	Gao et al., 2014, PMID: 25201530	Adeno
	MSKPCa7	Gao et al., 2014, PMID: 25201530	Adeno
	MSKPCa8	unpublished	Adeno
	MSKPCa9	unpublished	Adeno
	MSKPCa10	unpublished	NEPC
	MSKPCa11	unpublished	Adeno
	MSKPCa12	unpublished	Adeno
	MSKPCa13	unpublished	Adeno
	MSKPCa14	unpublished	NEPC
	MSKPCa15	unpublished	Adeno
	MSKPCa16	unpublished	NEPC
	MSKPCa17	unpublished	Adeno
	WCMC_PM154	Puca L. et al, 2018, PMID: 29921838	NEPC
	WCMC_PM155	Puca L. et al, 2018, PMID: 29921838	NEPC

Supplementary Table 5 (Related to Methods). Primer sequences used for qPCR experiments

**Oligos (qPCR)**	**Sequence (5'-3')**
MAP2 fw	CGAAGCGCCAATGGATTCC
MAP2 rv	TGAACTATCCTTGCAGACACCT
VGF fw	GGAACTGCGAGATTTCAGTCC
VGF rv	GTGCGGGTTTCCGTCTCTG
MTA1 fw	CATCAGAGGCCAACCTTTTCG
MTA1 rv	GCACGTATCTGTCGGTGGTC
SMARCC1 fw	TCTTGGGGCTGCTTACAAGTA
SMARCC1 rv	TCCATTCGAGATGGGTTCTGTAG
ACTB fw	TGACGTGGACATCCGCAAAG
ACTB rv	CTGGAAGGTGGACAGCGAGG
GAPDH fw	GACAGTCAGCCGCATCTTCT
GAPDH rv	TTAAAAGCAGCCCTGGTGAC
DPF1 fw	GTACAAGATCGACTGTGAAGCACC
DPF1 rv	CAACTGCTGTTTCTGACAGTCCATA
REST fw	GAACTCATACAGGAGAACGCCC
REST rv	GGCTTCTCACCTGAATGAGTACG
BAF53b fw	GAATGGCATGATCGAGGACTGGG
BAF53b rv	CGTGTGTTCCACGGAGCCTC
