# Supplementary material for: Role of specialized composition of SWI/SNF complexes in prostate cancer lineage plasticity
Source: Nat Commun. 2020 Nov 3;11:5549. doi: 10.1038/s41467-020-19328-1 (PMC7642293; doi:10.1038/s41467-020-19328-1)
Supplement: Supplementary file 3 — Description of Additional Supplementary Files [file 41467_2020_19328_MOESM3_ESM.pdf]

Description of Additional Supplementary Files

File Name: Supplementary Data 1
Description: Frequencies and statistical comparison (proportion test) of loss of heterozygosity alterations in SWI/SNF genes across PCa disease states.

File Name: Supplementary Data 2
Description: Frequencies of single nucleotide variation (SNV) mutations in SWI/SNF genes across PCa disease states.

File Name: Supplementary Data 3
Description: Frequencies of insertions/deletions (indels) in SWI/SNF genes across PCa disease states.

File Name: Supplementary Data 4
Description: Gene expression levels (assessed by RNA-seq) of SWI/SNF genes across PCa disease states: mean expression values and adjusted p-values (Mann-Whitney Wilcoxon test).

File Name: Supplementary Data 5
Description: Gene expression levels (RNA-seq) in LNCaP cells upon siRNA-mediated *SMARCA4* and *SMARCA2* knock-down (at 72 hours); Scrambled siRNA is used as control.

File Name: Supplementary Data 6
Description: Gene expression data (RNA-seq) in 22Rv1 cells upon siRNA-mediated *SMARCA4* and *SMARCA2* knock-down (at 72 hours); Scrambled siRNA is used as control.

File Name: Supplementary Data 7
Description: Mass spectrometry results for co-IP experiments using an anti-SMARCC1 (BAF155) antibody in NCI-H660 (CRPC-NE) cells, compared to an IgG isotype control using the iiTop3 analysis method. Results were obtained by analyzing three independent replicate experiments.

File Name: Supplementary Data 8
Description: Mass spectrometry results for co-IP experiments using an anti-SMARCC1 (BAF155) antibody in LNCaP-AR (adenocarcinoma) and NCI-H660 (CRPC-NE) cells, compared to an IgG isotype control using the iiTop3 analysis method. Results were obtained by analyzing three (NCI-H660) and two (LNCaP-AR) independent replicate experiments.

File Name: Supplementary Data 9
Description: Mass spectrometry results for co-IP experiments using an anti-SMARCA4 (BRG1) antibody in LNCaP (adenocarcinoma) and NCI-H660 (CRPC-NE) cells and an IgG isotype control. Results of two independent replicate experiments are shown.
