# Supplementary material for: Role of specialized composition of SWI/SNF complexes in prostate cancer lineage plasticity
Source: Nat Commun. 2020 Nov 3;11:5549. doi: 10.1038/s41467-020-19328-1 (PMC7642293; doi:10.1038/s41467-020-19328-1)
Supplement: Supplementary file 13 — Reporting Summary [file 41467_2020_19328_MOESM13_ESM.pdf]

nature research

Corresponding author(s): Rubin

Last updated by author(s): Sep 22, 2020

Reporting Summary

Nature Research wishes to improve the reproducibility of the work that we publish. This form provides structure for consistency and transparency in reporting. For further information on Nature Research policies, see Authors & Referees and the Editorial Policy Checklist.

Statistics

For all statistical analyses, confirm that the following items are present in the figure legend, table legend, main text, or Methods section.

n/a	Confirmed	
☐	☒	The exact sample size (*n*) for each experimental group/condition, given as a discrete number and unit of measurement
☐	☒	A statement on whether measurements were taken from distinct samples or whether the same sample was measured repeatedly
☐	☒	The statistical test(s) used AND whether they are one- or two-sided<br>*Only common tests should be described solely by name; describe more complex techniques in the Methods section.*
☐	☒	A description of all covariates tested
☐	☒	A description of any assumptions or corrections, such as tests of normality and adjustment for multiple comparisons
☐	☒	A full description of the statistical parameters including central tendency (e.g. means) or other basic estimates (e.g. regression coefficient) AND variation (e.g. standard deviation) or associated estimates of uncertainty (e.g. confidence intervals)
☐	☒	For null hypothesis testing, the test statistic (e.g. *F*, *t*, *r*) with confidence intervals, effect sizes, degrees of freedom and *P* value noted<br>*Give P values as exact values whenever suitable.*
☒	☐	For Bayesian analysis, information on the choice of priors and Markov chain Monte Carlo settings
☒	☐	For hierarchical and complex designs, identification of the appropriate level for tests and full reporting of outcomes
☐	☒	Estimates of effect sizes (e.g. Cohen's *d*, Pearson's *r*), indicating how they were calculated

Our web collection on statistics for biologists contains articles on many of the points above.

Software and code

Policy information about availability of computer code

Data collection	Non-commercial code: R-package will be provided upon request<br>MS: For differential expression testing the empirical Bayes test (R function EBayes from the limma package version 3.40.6)<br>RNA-seq: STAR v2.3.0e, RSEQtools and GENCODE v19 , TCGAbiolinks R package v2.12.2<br>Differential expression analysis: edgeR v3.28.1 package<br>Confluence analysis: IncuCyte S3 2018B software (v2018B)<br>single cell seq: SEURAT v. 3.1.5 package (PMID 29608179). GGPLOT2 3.3.0

Data analysis	Non-commercial code: R-package provided upon request.<br>-Survival analysis in the context of BRG1 and BRM expression : R (3.5.1) for Windows.<br>-MS data was interpreted with MaxQuant (version 1.6.1.0) against a SwissProt human database (release 2019_02) using the default MaxQuant settings. For differential expression testing the empirical Bayes test (R function EBayes from the limma package version 3.40.6) was performed on Top3 and LFQ protein intensities, using variance stabilization for the peptide normalization. The Benjamini and Hochberg method was further applied to correct for multiple testing.<br>-A Pearson correlation test was applied to test for correlations between SMARCA4/SMARCA2 and SOX2/SYP witrhin the MSK organoids.<br>- single cell analysis: data demultiplexing was performed using SEURAT v. 3.1.5 package (PMID 29608179). Low quality cells and multiplets were excluded by removing cells with unique feature counts over 5500 or less than 1000. Cells containing mitochondrial gene counts greater than 25% were also removed. Data were then scaled to 10,000 and log transformed. Only cells expressing SOX2 and SMARCA4 genes were included. Boxplots were drawn using GGPLOT2 3.3.0 (https://ggplot2.tidyverse.org) and p-values were calculated using Wilcoxon test.<br>-CHIP-seq: Peak comparison was performed using BEDTOOLS v2.29.0 (https://bedtools.readthedocs.io/en/latest/#).<br>CHIP-seq: ChIP-seq peaks for SMARCC1 and HOXB13 in LNCaP cells were downloaded from GEO: GSE110655 [https://www.ncbi.nlm.nih.gov/geo/query/acc.cgi?acc=GSE110655] and GSE94682 [https://www.ncbi.nlm.nih.gov/geo/query/acc.cgi?acc=GSE94682], respectively. ChIP-seq peaks for H3K27ac and H3K27me3 in LNCaP cells were from data published by Sandoval et al.

For manuscripts utilizing custom algorithms or software that are central to the research but not yet described in published literature, software must be made available to editors/reviewers. We strongly encourage code deposition in a community repository (e.g. GitHub). See the Nature Research guidelines for submitting code & software for further information.

Data

Policy information about availability of data

All manuscripts must include a data availability statement. This statement should provide the following information, where applicable:

- Accession codes, unique identifiers, or web links for publicly available datasets
- A list of figures that have associated raw data
- A description of any restrictions on data availability

Data have been submitted on the European Genome-phenome Archive under the accession EGAS00001004177.
The mass spectrometry proteomics data have been deposited to the ProteomeXchange Consortium via the PRIDE103 database with the dataset identifier PXD016861.

Field-specific reporting

Please select the one below that is the best fit for your research. If you are not sure, read the appropriate sections before making your selection.

☒ Life sciences ☐ Behavioural & social sciences ☐ Ecological, evolutionary & environmental sciences

For a reference copy of the document with all sections, see nature.com/documents/nr-reporting-summary-flat.pdf

Life sciences study design

All studies must disclose on these points even when the disclosure is negative.

Sample size	No sample size calculation was performed. Sample size was based on the available cohorts and was sufficient for the analyses performed (for cox proportional hazards modeling, the number of covariates added to the models were restricted to reduced over-fitting).
Data exclusions	No subjects were removed from the analysis presented.<br>In the analysis of immunostainings presented in Fig.1d, slight discrepancies in sample sizes between different markers are due a fraction of samples for each marker that could not be evaluated due to technical reasons (such as tissue core missing on the TMA).
Replication	Knock-down experiments, cell growth experiments, RNA-sequencing, qPCR and mass spectrometry were performed in triplicates or in duplicates, as specified in Figure legends and Supplementary legends. Each replicate represents an independent experiment performed at a different time. All statistical analyses and figures pertain to pooled results from all these replicates.
Randomization	This is not relevant to our study (analyses compared groups that were defined by their phenotypes). CHIP-seq: ChIP-seq peaks for SMARCC1 and HOXB13 in LNCaP cells were downloaded from GEO: GSE110655 and GSE94682, respectively. ChIP-seq peaks for H3K27ac and H3K27me3 in LNCaP cells were from data published by Sandoval et al.
Blinding	Immunohistochemistry on tissue microarrays (TMA) and whole slides was scored by a pathologist blinded to clinical or diagnostic information. For assessment of BRG1 and BRM immunostainings in the context of patient outcomes, the scoring of scanned slides was performed using a TMA scoring tool Scorenado (University of Bern, Bern, Switzerland), which presents TMA spots in random order and without any accompanying information to the pathologist scoring the stainings.

Reporting for specific materials, systems and methods

We require information from authors about some types of materials, experimental systems and methods used in many studies. Here, indicate whether each material, system or method listed is relevant to your study. If you are not sure if a list item applies to your research, read the appropriate section before selecting a response.

Materials & experimental systems

n/a	Involved in the study	
☐	☒	Antibodies
☐	☒	Eukaryotic cell lines
☒	☐	Palaeontology
☒	☐	Animals and other organisms
☒	☐	Human research participants
☐	☒	Clinical data

Methods

n/a	Involved in the study	
☐	☒	ChIP-seq
☒	☐	Flow cytometry
☒	☐	MRI-based neuroimaging

Antibodies

Antibodies used

AR Abcam ER179(2) ab108341
BAF155 Abcam EPR12395 ab172638
BAF170 Cell Signaling Technology D8O9V 12760
BAF45B Atlas Antibodies polyclonal HPA049148
BAF47 (INI-1) BD Biosciences bd612110
BAF47 (INI-1) Abcam EPR12014 ab181976
BAF53A Abcam EPR7443 ab131272
BAF53B Abcam EP10101 ab180927
Brg1 Abcam EPR3912 ab108318
Brm Cell Signaling Technology D9E8B 11966
EZH2 Active Motif polyclonal 39933
GAPDH Millipore Sigma polyclonal AB2302
Histone H3 Cell Signaling Technology 4499
HOXB13 Novus NP2-48778
Ki-67 Dako MIB-1 M7240
MAP2 Abcam ab32454
Mouse Anti-rabbit IgG (Conformation specific) Cell Signaling Technology 5127
MTA1 Cell Signaling Technology D40DY 5647
NKX3.1 Cell Signaling Technology D2Y1A 83700
p-Rb1 Cell Signaling Technology D20B12 8516
p21 Cell Signaling Technology 12D1 2947
p53 Santa Cruz DO-1 sc-126
rabbit IgG Isotype Control antibody . Thermo fisher Scientific, 026102
Rb1 Abcam E182 ab32513
REST Millipore Sigma polyclonal 07-579
SOX2 Cell Signaling Technology D6D9 3579
synaptophysin Thermo Scientific SP11 RM9111-S
synaptophysin Abcam YE269 ab32127
TTF1 / NKX2.1 Abcam EP1584Y ab76013
VGF Abcam ab69989
Vinculin Abcam EPR8185 ab129002

Validation

Abcam validation : https://www.abcam.com/primary-antibodies/how-we-validate-our-antibodies
Western blot
Antibodies are validated in western blot using lysates from cells or tissues that we have identified to express the protein of interest. Once we have determined the right lysates to use, western blots are run and the band size is checked for the expected molecular weight. We will always run several controls in the same western blot experiment, including positive lysate and negative lysate (if possible, Figure 2).

When possible, we also include knock-out (KO) cell lines as a true negative control for our western blots. We are always increasing the number of KO-validated antibodies we provide. In addition, we run old stock alongside our new stock. If we know the old stock works well, this also acts as a suitable positive control.

If the western blot result gives a clear clean band and we are happy with the result from the control lanes, these antibodies will be passed and added to the catalog
Cell signalling validation policies: https://www.cellsignal.com/contents/our-approach/cst-antibody-validation-principles/ourapproach-validation-principles

IP
When we validate antibodies for IP, we carry out a standard IP protocol using magnetic beads as we find that these beads give better results in less time. Once we have isolated our protein of interest we run the supernatant alongside the flow-through from the IP on a western blot. We also run a no-antibody and bead-only control for each lysate we test. which we run on the same western blot. When we do this, we are looking for a clean band at the expected protein size from our IP sample. We are also checking the other lanes of the blot to check for non-specific binding that would appear also in our negative controls.

Immunohistochemistry and immunocytochemistry
IHC and ICC determine whether an antibody recognizes the correct protein based on cellular and subcellular localization.

Antibody specificity is confirmed by looking at cells that either do or do not express the target protein within the same tissue. Initially, our scientists will review the available literature to determine the best cell lines and tissues to use for validation. We then check the protein expression by IHC/ICC to see if it has the expected cellular localization (Figure 3). If the localization of the signal is as expected, this antibody will pass and is considered suitable for use in IHC/ICC.

Active Motif : https://www.activemotif.com/antibody-development
Immunogen Selection
Immunogens are selected to decrease the likelihood of cross-reactivity with related proteins and to maximize detection of the protein in its native context. Immunogens for modification-specific antibodies are selected to ensure that the antibody recognizes the modified version of the protein only and does not cross-react with the same modification on different proteins.
Specificity screening
The first test performed on every antibody is dot blot analysis, which ensures its specificity for the desired protein or modification. Antibodies that do not exhibit a greater than 25-fold selectivity for the desired modification are failed.
Peptide array validation
For histone modification antibodies that require more stringent specificty testing, we utilize our MODified™ Histone Peptide Array to identify and quantitate any cross-reactivity to other modifications. The MODified Histone Peptide Arrays screen 59 modifications on the N-terminal tails of histones H2A, H2B, H3 and H4. Active Motif is the only company that performs this additional level of testing.
Western Blot
Western blotting is performed to verify the antibody recognizes a protein of the correct molecular weight and does not cross-react with other proteins. Commonly used chemical treatments that stimulate a specific modification (e.g. HDAC inhibitors and acetylation) are used to better detect the modification.
Technique Validation
Each antibody is validated for use in important techniques such as chromatin immunoprecipitation (ChIP) and immunofluorescence (IF), so that you have confidence when using them in your experiments.

Santa Cruz: https://www.labome.com/method/Santa-Cruz-Antibodies.html
p53 (DO-1): sc-126. Western blot analysis of p53 expression in SW480 (A), A549 (B) and HUV-EC-C (C) whole cell lysates.
our product anti-p53 Antibody (DO-1) is a mouse monoclonal IgG2a (kappa light chain) p53 antibody provided under the catalog number sc-126. It is available in various formats including directly conjugated to Agarose, HRP, Phycoerythrin, FITC, Alexa Fluor® 405, Alexa Fluor® 488, Alexa Fluor® 546, Alexa Fluor® 594, Alexa Fluor® 647, Alexa Fluor® 680, Alexa Fluor® 790, TRITC, PerCP, PerCP-Cy5.5 and also as TransCruz reagent sc-126 X.
This clone is a well established reagent and we currently list >5.059 scientific publications including a number of high impact factor publications referring to the good use of this antibody since more than two decades. Product citations also include the use of this antibody together with the also offered CRISPR KO plasmid to confirm the successful p53 Knock-Out. Corresponding citations can be found on our product website for sc-416469.
Quality assurance measures are taken throughout the manufacturing and purification process to assure the highest quality reagent is produced. This antibody sc-126 has been used on various Sample materials and different species and it is recommended for a variety of applications including WB, IP, IF, IHC(P) and FCM. Control experiments carried out to confirm reactivity and specificity include e.g. ELISA against recomb. p53 protein fragments, FCM, IHC(P) and detection of p53 activation, p53 gene Silencing, Knock-Out, WB with cells transfected with p53 and simultaneous Western Blot on endogenous p53 to confirm the correct protein localization, protein size and protein expression patterns. A few sample images of experiments carried out are shown on our website. These include e.g. Simultaneous direct near-infrared western blot analysis of p53 expression, detected with p53 (DO-1) AF680: sc-126 AF680 and GAPDH expression, detected with GAPDH (0411) Alexa Fluor® 790: sc-47724 AF790 in A-431 (A) and SW480 (B) whole cell lysates. Blocked with UltraCruz® Blocking Reagent: sc-516214.
ect.
Additional details and more data images can be found on the corresponding product website and the datasheet provided for download for sc-126.
However, of course we always also strongly suggest that every researcher carefully does set-up their own appropriate positive and negative controls suitable for their specific individual experiment for all reagents used, including the 1° and 2° antibodies. This is strongly recommended and good scientific practice of course to verify the results obtained and in order to being able interpret the data obtained in the individual setting.

Merck Millipore: https://www.merckmillipore.com/CH/de/life-science-research/antibodies-assays/antibodies-overview/Antibody-Development-and-Validation/cFOb.qB.8McAAAFOb64qQvSS,nav

At Merck, our commitment to advancing scientific research defines us
Our commitment to produce quality antibodies is based on innovation, customer beta testing and feedback to the design/engineering team. These efforts and collaborations have led to new validation techniques and novel antibody-based technologies, such as improved bead-based multiplex assays and imaging flow cytometry.

anti-REST 07-579
: Quality Control Testing

Evaluated by western blot with ES cell total extract. Western Blot Analysis: 1:500-1:2000 dilution of this antibody detected REST in ES cell total extracts.

Eukaryotic cell lines

Policy information about cell lines

Cell line source(s)

Commercially available PCa cell lines (RWPE-1, LNCaP, 22Rv1, VCaP, LAPC4, PC3, DU145, NCI-H660, C4-2) were purchased from ATCC.

	WCM154 and WCM155 CRPC-NE cell lines have been previously established (doi: 10.1038/s41467-018-04495-z)<br><br>LNCaP-AR cells were a kind gift from Dr. Sawyers and Dr. Mu (Memorial Sloan Kettering Cancer Center) and were previously used in published studies (doi: 10.1126/science.aah4307).<br><br>All MSK-PCa cell lines (CRPC-Adeno and CRPC-NE) were a kind gift from Dr. Chen (Memorial Sloan Kettering Cancer Center) and previously published (doi: 10.1016/j.cell.2014.08.016).
Authentication	Cell Line Authentication Service - Microsynth (https://www.microsynth.ch/cell-line-authentication.html#HowItWorks) once a year by STR profiling.
Mycoplasma contamination	Cell cultures were regularly (every 3 months) tested for Mycoplasma contamination and confirmed to be negative.
Commonly misidentified lines<br>(See ICLAC register)	no commonly misidentified lines were used in this study

Clinical data

Policy information about clinical studies
All manuscripts should comply with the ICMJE guidelines for publication of clinical research and a completed CONSORT checklist must be included with all submissions.

Clinical trial registration	clinical data for the validation of the SMARCA4 knock-down signature is publicly available and was downloaded from<br>1) The Cancer Genome Atlas (TCGA)<br>2) Abida et al. (doi: 10.1073/pnas.1902651116)<br>3) Beltran et al. doi: 10.1038/nm.4045
Study protocol	*Note where the full trial protocol can be accessed OR if not available, explain why.*
Data collection	*Describe the settings and locales of data collection, noting the time periods of recruitment and data collection.*
Outcomes	*Describe how you pre-defined primary and secondary outcome measures and how you assessed these measures.*

ChIP-seq

Data deposition

☐ Confirm that both raw and final processed data have been deposited in a public database such as GEO.

☐ Confirm that you have deposited or provided access to graph files (e.g. BED files) for the called peaks.

Data access links<br>*May remain private before publication.*	*For "Initial submission" or "Revised version" documents, provide reviewer access links. For your "Final submission" document, provide a link to the deposited data.*
Files in database submission	*Provide a list of all files available in the database submission.*
Genome browser session<br>(e.g. UCSC)	*Provide a link to an anonymized genome browser session for "Initial submission" and "Revised version" documents only, to enable peer review. Write "no longer applicable" for "Final submission" documents.*

Methodology

Replicates	*Describe the experimental replicates, specifying number, type and replicate agreement.*
Sequencing depth	*Describe the sequencing depth for each experiment, providing the total number of reads, uniquely mapped reads, length of reads and whether they were paired- or single-end.*
Antibodies	*Describe the antibodies used for the ChIP-seq experiments; as applicable, provide supplier name, catalog number, clone name, and lot number.*
Peak calling parameters	*Specify the command line program and parameters used for read mapping and peak calling, including the ChIP, control and index files used.*
Data quality	*Describe the methods used to ensure data quality in full detail, including how many peaks are at FDR 5% and above 5-fold enrichment.*
Software	*Describe the software used to collect and analyze the ChIP-seq data. For custom code that has been deposited into a community repository, provide accession details.*
